# Gestalt laws enhance the representation of figures over backgrounds in the visual cortex and influence contrast perception

**DOI:** 10.1038/s41598-026-45730-8

**Published:** 2026-04-07

**Authors:** Anne F. van Ham, Danique Jeurissen, Matthew W. Self, Pieter R. Roelfsema

**Affiliations:** 1 Department of Vision and Cognition, Netherlands Institute for Neuroscience, Meibergdreef 47, Amsterdam, 1105 BA the Netherlands; 2 Center for Neural Science, New York University, 4 Washington Pl, New York, NY USA; 3 School of Psychology and Neuroscience, University of Glasgow, Glasgow, Scotland; 4 Department of Integrative Neurophysiology, Center for Neurogenomics and Cognitive Research, VU University, De Boelelaan 1085, Amsterdam, The Netherlands; 5 Department of Neurosurgery, Academic Medical Center, Amsterdam, The Netherlands; 6 Laboratory of Visual Brain Therapy, Sorbonne Université, Institut National de la Santé et de la Recherche Médicale, Centre National de la Recherche Scientifique, Institut de la Vision, Paris, 75012 France

**Keywords:** Perceptual grouping, Figure-background modulation, Gestalt laws, Contrast discrimination, Visual cortex, Feedback projections, Primates., Neuroscience, Psychology

## Abstract

The Gestalt laws of perceptual organization determine which image regions in a visual scene are perceived as figures or background. Convex, enclosed, and symmetric regions are more likely to be perceived as figures, but the underlying neural mechanisms are not well understood. Here, we studied the influence of Gestalt cues on visual perception and on neuronal activity in early visual cortex. We found that, in both humans and macaques, the perceived contrast of Gabor probes was generally higher when placed on Gestalt-defined figures than on background regions, although individuals differed. Neural activity in areas V1 and V4 of the visual cortex of monkeys performing a contrast discrimination task was higher for image elements belonging to Gestalt-defined figures than image-elements of the background, except for symmetry-defined figures. We observed this figure-background modulation in both V1 and V4 even when the distinction between figure and background was irrelevant for the task of the animal. The influence of Gestalt cues on neural activity was stronger in V4 than in V1, suggesting that Gestalt organization arises through modulation of neural activity in higher visual areas, which then modulates neural activity in early visual areas through feedback.

**Supplementary Information:**

The online version contains supplementary material available at 10.1038/s41598-026-45730-8.

## Introduction

The visual system initially analyzes the visual scene in a piece-meal manner. Neurons at early levels of the visual pathways perform a local analysis of the image elements falling in their small receptive fields (RFs). The visual system needs to determine which of these image elements belong to the same objects and which belong to other objects and the background. Perceptual organization is the process by which the visual system groups the image elements that belong to coherent objects and segregates them from other objects and the background. In human vision, this process of perceptual organization is highly efficient, and we can effortlessly determine where in the image an object ends and another one begins. The Gestalt laws describe principles that influence perceptual organization^1–4^. Some of the Gestalt cues determine the grouping of image elements. For instance, image elements that are similar, nearby and in each other’s good continuation usually belong to the same object. When a subject directs attention to a particular image element, visual cortical neurons that encode the attended image element enhance their response, and this response enhancement spreads to other items that are part of the same perceptual group^5^. Other Gestalt cues influence the perception of figure and background surfaces: image regions that are small, symmetric, enclosed and convex are more likely to be perceived as figures than image regions that are not^3,6–9^. In the present study, we focus on these latter Gestalt cues that determine ‘figureness’.

**Fig. 1:**
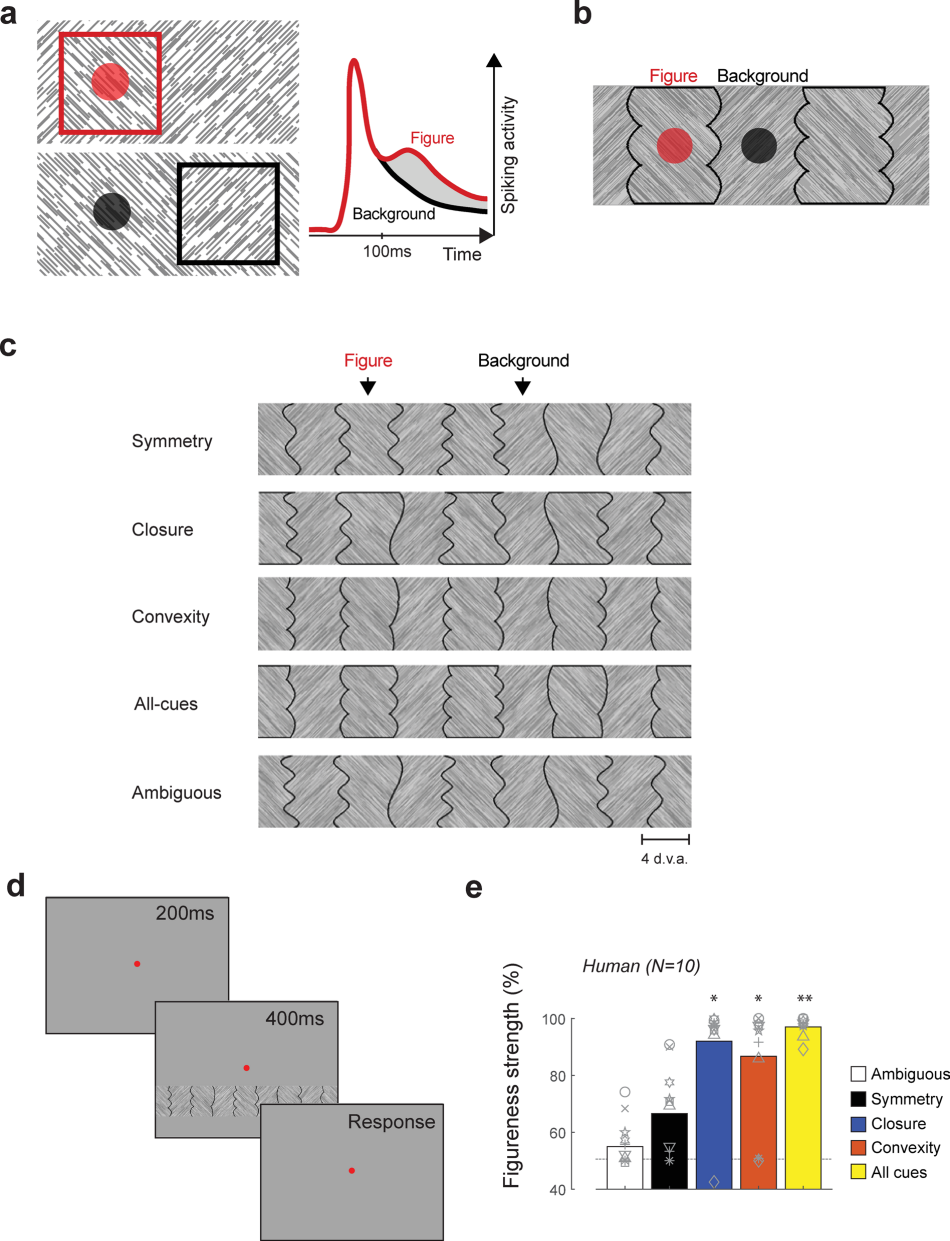
Stimuli and task. (**a**) left, Previous studies used size differences to determine figure-background organization (e.g., Lamme, 1995, Zipser et al., 1996): the smaller square region is perceived as a figure on top of a background. The colored circles indicate typical V1 RF sizes. right, Schematic indicating that the firing rate of V1 neurons is higher when their RF falls on the figure than on the background, an effect known as figure-background modulation (FBM – gray region). (**b**) The Gestalt cues of symmetry, closure, and convexity establish a figure percept even when background regions have the same size. (**c**) Gestalt cues were tested individually (top three rows) and combined (fourth row). We used a stimulus (fifth row) for which figure-background organization was ambiguous for comparison. Note that the orientation of the texture of the image regions alternates and that they are separated by black edges. (**d**) Human participants judged whether the image region directly below the fixation point was perceived as a figure or background. (**e**) The average percentage of trials on which participants judged symmetric, closed and convex regions to be figural. Grey symbols indicate individual participants. **, *p* < 0.01, *, *p* < 0.05 (Wilcoxon signed rank test, Bonferroni corrected). Dashed line, chance level (50%).

The neural mechanisms by which Gestalt cues influence figure-background perception are only partially understood. A well-studied Gestalt law is that of size: small image regions are usually perceived as figures and larger regions as background. This can be seen in Fig. 1a (left panel), where the smaller region with image elements of one orientation is perceived as a square figure, superimposed on a background with elements of the opposite orientation. The firing rate of neurons in the primary visual cortex (V1) is higher if their receptive field (RF) falls on a figure than if it falls on the background (Fig. 1a)^10,11^, an effect known as figure-background modulation (FBM). FBM arises through recurrent interactions between neurons in V1 and higher cortical areas^12,13^.

Along the visual hierarchy, tuning of neurons becomes more complex, from points of lights to orientation, shape, and entire objects^14,15^. Neurons in mid-tier and higher areas, such as V4 and inferior temporal cortex (IT), encode object shape and have large RFs^16,17^. These areas contain neurons which encode the curvature and shape of contours in a translation invariant manner^18–22^, and the initial responses of neurons in V4 have been shown to reflect figure-background structure in both artificial^23,24^ and natural images^25^.

It is conceivable that the Gestalt rules such as symmetry, convexity and closure impact on the RF tuning properties of cells at these higher levels. These areas can modulate V1 activity through feedback projections, which might cause late modulatory influences. In line with this idea, early V1 activity (< 100ms) represents the local features in the neuron’s RF, later activity (100-120ms) reflects an initial estimate of figure-background relationships based on nearby contextual influences, and activity finally (> 120ms) evolves to a pattern in which the global scene layout is taken into account and the entire figure representation is labeled with enhanced neuronal activity^26^. The idea that FBM arises through recurrent connections between V1 and the higher visual areas is also supported by the fact that FBM is weaker when the subject does not pay attention to the figure^24^ and when neuronal activity in higher visual areas is perturbed^13,27^.

In almost all previous studies on the neural mechanisms of figure-background organization, the figure was smaller than the background, which raises the possibility of alternative explanations for the pattern of FBM. The large uniform surround used in these studies produces strong suppression of neural activity^28,29^ and theoretical studies related this suppression to predictive coding^30^. Furthermore, in most previous neurophysiological studies on figure-background segregation the animal was cued to attend the figure, either because it had to make an eye movement towards it^10–12^ or a perceptual judgement about its shape^26^. In the present study, we tested whether the Gestalt cues of symmetry, closure and convexity can also induce FBM in early visual areas when figure and background are matched in size (Fig. 1b). We used a task in which figure-background organization was irrelevant, avoiding the effects of visual attention. As an indirect measure of figure-background segregation, we measured the effect of the Gestalt cues on the perception of the contrast of a Gabor probe because previous studies have demonstrated that the perceived contrast (PC) of image elements is higher on figures than on the background^31^.

We first validated that our stimuli produced perceptual segregation in humans by measuring perceived contrast on figures and backgrounds using a previously established method^31^. On average, the perceived contrast was higher on figures than on backgrounds. We then examined neural activity in V1 and V4 of macaque monkeys performing the same contrast task and found that shapes defined by closure and convexity induce FBM. The effect of symmetry on both contrast perception and FBM was weak. Combining all three Gestalt cues produced stronger neural modulation than individual Gestalt cues in isolation. Interestingly, FBM in V4 was stronger than in V1, suggesting that feedback from higher to lower visual cortical areas induces FBM in V1. These results generalize our understanding about how figures are segregated from their background, by showing how the Gestalt laws influence figure-background and contrast perception and elicit FBM in early visual areas.

## Results

### Gestalt cues influence figure-background perception

We first examined influence of different Gestalt cues on figure-background perception in humans. We created texture strips with regions of alternating orientation (Fig. 1b, c, see Methods). Adjacent image regions were either symmetric or asymmetric, closed or open, convex or concave (as in^32^ or all cues were combined. The width of the image regions was approximately 5 deg. We asked ten human observers to report whether they perceived the region presented directly below the fixation point as figure or background (Fig. 1d). On average only 66.5% of symmetrical regions were perceived as figures across participants, an effect that did not differ significantly from chance level (chance level = 50%, Wilcoxon signed rank test, *p* = 0.1, Bonferroni corrected) (Fig. 1e). The other cues biased figure-background perception, because 92% of the enclosed regions and 87% of the convex regions were perceived as figures, and when all cues were combined 97% of the symmetrical, closed and convex image regions were perceived as figures. As a control we also tested ambiguous regions and observed no significant effect (*p* = 0.5), which indicates the absence of a bias in figure perception for the position below the fixation point. Hence, closure and convexity induced figural percepts in our stimulus set but the influence of symmetry was weak for this stimulus configuration.

### Gestalt cues influence contrast perception

#### Humans

**Fig. 2:**
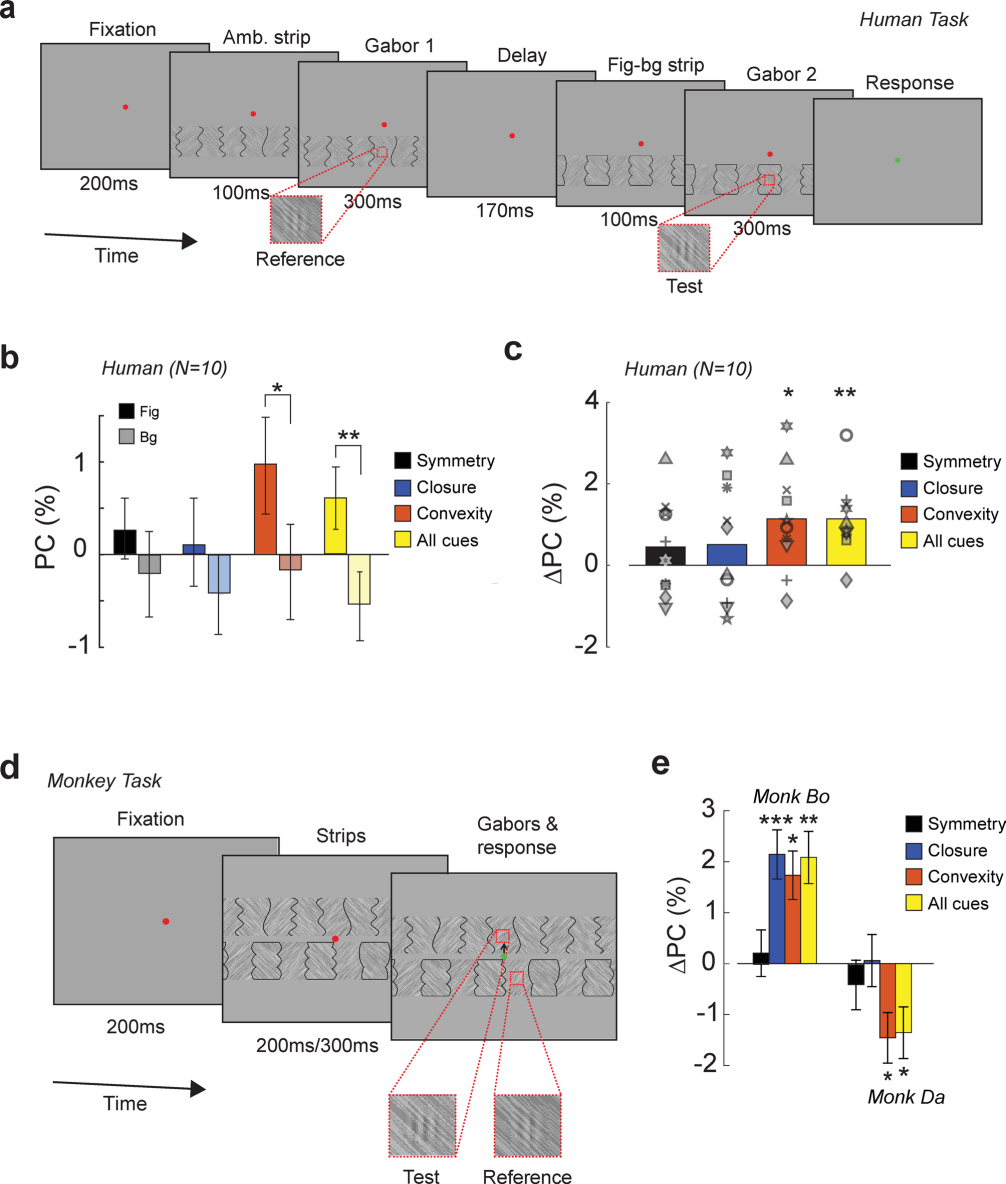
The influence of Gestalt cues on contrast perception. (**a**) Humans performed a temporal 2-alternative forced choice (t2AFC) contrast discrimination task, judging which of two embedded Gabor targets was higher in contrast. (**b**) Average perceived contrast difference (PC) between the reference Gabor situated on an ambiguous strip and a test Gabor stimulus situated on a figure (darker colors) or background (lighter colors) region per Gestalt cue (symmetry = black, convexity = orange, closure = blue, All cues = yellow). PC was higher for Gabors on figures than on backgrounds. (**c**) Same data as in b, but now the PC difference between figures and backgrounds is shown. The symbols denote individual participants. (**d**) Spatial 2AFC contrast discrimination task for monkeys. The monkeys had to make an eye movement to the Gabor that was highest in contrast. (**e**) PC difference between figures and backgrounds in monkey Bo and Da. Monkey Bo perceived Gabors situated on figures were perceived as higher in contrast than Gabors on backgrounds (except for symmetry-defined figures). In monkey Da, the pattern was reversed, Gabors on background regions were perceived as higher in contrast. Error bars in all panels are 1 s.e.m. *, *p* < 0.05, **, *p* < 0.01, ***, *p* < 0.001.

We aimed to measure FBM without the confounding effects of attentional selection, which enhances neural activity in visual areas and could obscure the effects of figure-background segregation. We therefore used an indirect temporal two-alternative forced choice task to infer figure-background segregation strength that did not require subjects to report about the figure regions. A previous study^31^, demonstrated that the perceived contrast of Gabor probes is higher on small figures compared to a larger background region, using stimuli similar to those in Fig. 1a. We now used this contrast judgement task to infer the strength of figure-background segregation for the strips with embedded Gestalt cues of Fig. 1c. Ten human participants indicated which of two Gabor probes was perceived as higher in contrast. A reference Gabor element was presented on an ambiguous strip and a test Gabor element on a strip containing one or all Gestalt cues. The two strips were presented successively (Fig. 2a). To measure perceived contrast (PC), we estimated the contrast of the test Gabor (with variable contrast) that gave rise to an accuracy of 75% with a staircase procedure (see Methods). On average, PC was higher on figural regions than on background regions if the Gestalt cue was convexity and also if all cues were combined (Fig. 2b), and we determined the difference in PC between these conditions, ΔPC, for every subject (Fig. 2c). The ΔPC for convexity was 1.1% because the PC of Gabors on convex image regions was higher than that on concave regions. Eight of the ten participants had a PC that was higher on convex figures but there were also two subjects with an opposite effect (Fig. 2c). Despite this inter-individual variability, convexity increased PC when all subjects were analyzed together (paired t-test, *t*_*9*_ = 2.8, *p* = 0.02). We obtained similar results when all Gestalt cues were combined: nine of the ten participants showed higher PC on figures than on backgrounds, with an average increase of 1.1% PC (*t*_*9*_ = 3.9, *p* = 0.004). The influence of closure and symmetry on PC was weaker and failed to reach significance (*t*_*9*_ = 1.1, *p* = 0.30 and *t*_*9*_ = 1.2, *p* = 0.25, respectively). Interestingly, there was a correlation between the influence of figure-background perception on PC across conditions (Intraclass correlation of rankings, correlation coefficient = 0.29, F_9,30_ = 2.7, *p* = 0.02). Hence the influence of one Gestalt cue on PC for a particular participant predicted the influence of another Gestalt cue on PC for the same participant.

#### Monkeys

We next examined the influence of the Gestalt cues on contrast perception in three macaque monkeys, Bo, Du and Da. Here, we used a spatial two-alternative forced choice task which is more suitable for monkeys as it requires a shorter trial duration and is easier to train than a temporal forced-choice task. We simultaneously presented the ambiguous strip and the figure-background strip above and below fixation. Now the test Gabor was presented on the ambiguous strip and the reference Gabor (constant contrast) on the Gestalt strip (Fig. 2d). The monkeys’ task was to make an eye movement to the Gabor with the higher contrast. Monkeys Bo and Da performed the task well, but monkey Du developed a strong bias for choosing the Gabor on the lower strip, resulting in low accuracy so that we could not measure the PC of the reference Gabor for this monkey. The pattern of PC values differed between monkey Bo and Da. In monkey Bo, the pattern of PCs closely resembled that in humans. PC was higher on figures than on backgrounds for closed (bootstrap test, *p* < 0.001) and convex image regions (*p* = 0.049) and in the all-cues condition (*p* = 0.002), but the effect of symmetry was weak (*p* = 0.7) (Fig. 2e). In contrast, in monkey Da, PC was generally lower on figures than backgrounds, with a lower PC for convexity (*p* = 0.03) and all cues combined (*p* = 0.04). Although the results were reversed for this monkey, the ordering of effects was similar to those in humans, with the strongest effects for convexity and the all-cues condition, and weaker results for symmetry-defined figures. This difference between monkeys Bo and Da replicates the inter-individual differences and the correlation across conditions of the human observers. The results of Monkey Bo resembled those of most human subjects who perceived a higher contrast on figures and the results of Monkey Da resembled those of the more exceptional subjects perceiving a higher contrast on the ground. A later section will show how these differences reflect distinct interactions between the influence of Gestalt cues and the Gabor element on the neuronal responses, providing insight into the cause of this interindividual variability.

### The influence of Gestalt cues on neural activity in V1

We next examined if the figure-background organization induced by Gestalt cues influenced multi-unit spiking activity (MUA) in areas V1 and V4, recorded with chronically implanted Utah micro-electrode arrays. We recorded neural activity while the monkeys performed the contrast discrimination task in which the figure-background structure was irrelevant (Fig. 2d). We included a total of 111 recording sites in area V1 (19 in monkey Bo, 67 in monkey Du and 25 in monkey Da) and 75 sites in V4 (32 in monkey Bo, 29 in monkey Du and 14 in monkey Da). We compared activity in response to the image regions which, according to the Gestalt cues, should be interpreted as figure or background. The textured strip was positioned on a given trial such that either a figural or background region fell on the center of the aggregate RF of one of the electrode arrays (Supplementary Figure S1). The local elements within the V1 RFs were identical on figure and background regions so that any difference in neuronal activity between conditions is a contextual influence, from outside the RF. We analyzed the neural responses in the epoch during which the monkeys had their gaze at the fixation point, ensuring that there was no influence of eye movements on the neural response.

**Fig. 3:**
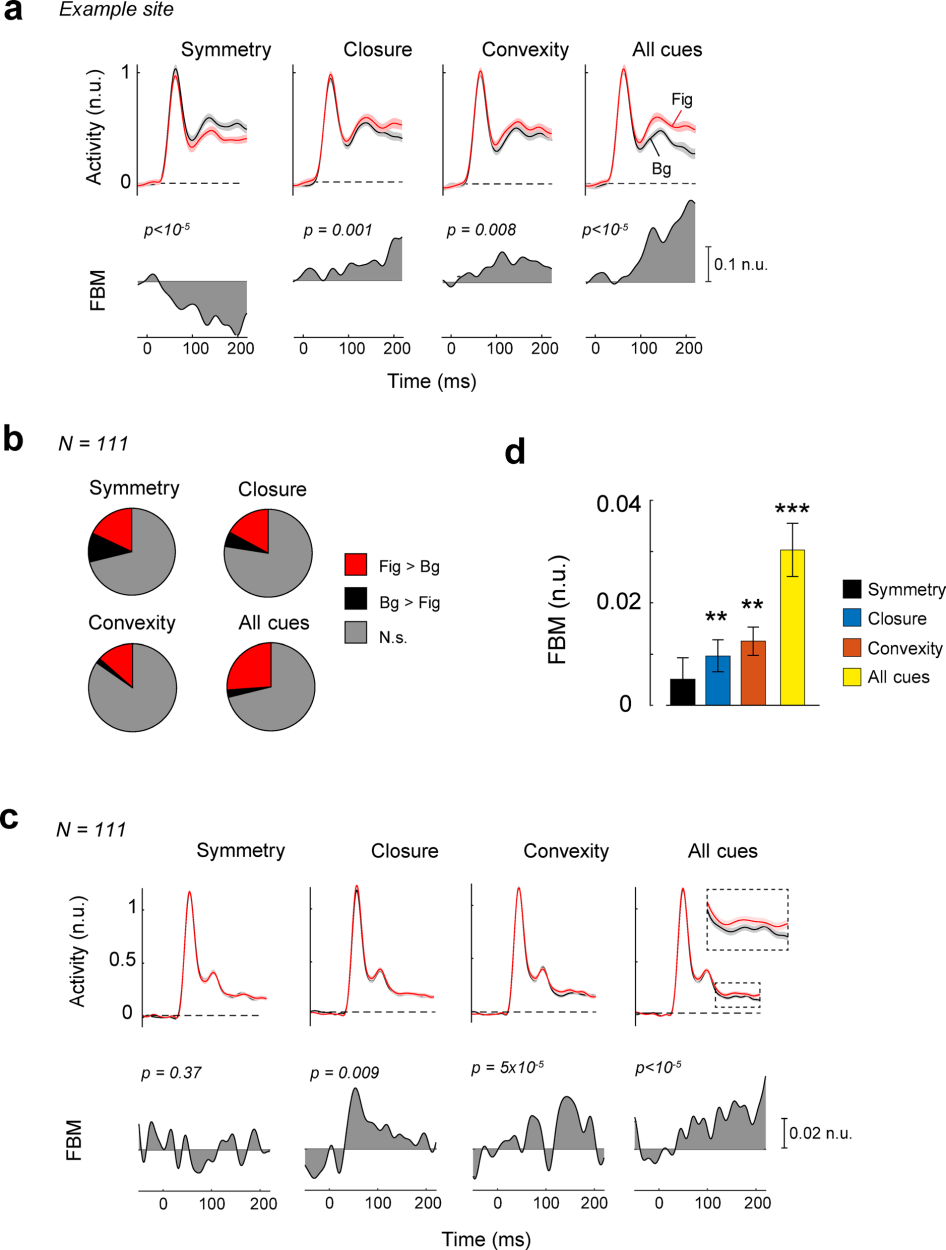
FBM in V1. (**a**) Neuronal activity at an example V1 recording site from monkey Bo elicited by figural (red) and background regions (black). n.u. = normalized units, activity normalized to the peak of the transient response. The shaded area represents ± 1 s.e.m. across trials. Lower panels, response difference between figural and background regions. (**b**) The proportion of recording sites with a significant (*p* < 0.05) enhancement (red) or suppression (black) of activity by figural regions. Grey, absence of a significant effect. (**c**) The average V1 response evoked by figural (red) and background regions (black). Lower panels, response difference between figural and background regions (**d**) The average FBM across V1 sites for different Gestalt cues (symmetry = black, convexity = orange, closure = blue, All cues = yellow) in n.u., i.e. compared to the peak response. Error bars represent s.e.m. **, *p* < 0.01 (Bonferroni corrected), ***, *p* < 0.001.

In area V1, we only included recording sites for which the RF did not overlap with the edges between the image regions, which had a width of 5 d.v.a. (see Methods). The figural regions generally elicited stronger activity than background regions (Fig. 3). Figure 3a illustrates an example V1 recording site at which the neurons responded more vigorously to closed (independent samples t-test, t_942_ = 3.2, *p* = 0.0013, time window from 100-240ms) and convex image regions (t_937_ = 2.6. *p* = 0.008) than to open and concave regions. We observed the same effect when all cues were combined (t_936_ = 7.8, *p* < 10^− 5^), but, unexpectedly, asymmetric regions elicited stronger activity than symmetric ones (t_940_ = -4.6, *p* < 10^− 5^). Figure 3b shows the proportion of recording sites that exhibited a significant difference in activity between figures and backgrounds, for each of the Gestalt cues. The influence of perceptual organization was most pronounced if all Gestalt cues were combined. In this condition 26% of the V1 recording sites exhibited a significant effect.

We averaged responses across all V1 recording sites (Fig. 3c) and observed that closed (t_110_ = 3.1, *p* = 0.009, Bonferroni corrected) and convex image regions (t_110_ = 4.6, *p* = 5 × 10^− 5^) elicited more V1 activity, on average, than open and concave regions, and the same effect was observed when all cues were combined (t_110_ = 5.8, *p* < 10^− 5^). The effect in V1 was relatively small, and varied across monkeys in V1 (Supplementary Figures S2, S3). We did not observe a significant effect of symmetry on the average V1 response (t_110_ = 0.90, *p* = 0.37). A weighted linear model revealed that the magnitude of FBM differed significantly between conditions (F_3,330_ = 7.3, *p* = 9 × 10^− 5^) (Fig. 3c) because FBM for stimuli with combined Gestalt cues was strongest (post-hoc test, *p* < 0.01, Bonferroni corrected) (Fig. 3d). Our design did not require the monkeys to attend to the figural regions and, accordingly, the influence of the Gestalt cues on V1 activity was weaker than in previous studies in which monkeys reported the shape or location of the figure^10,11,24,26,33^.

### Modulation of V4 activity by Gestalt cues

**Fig. 4:**
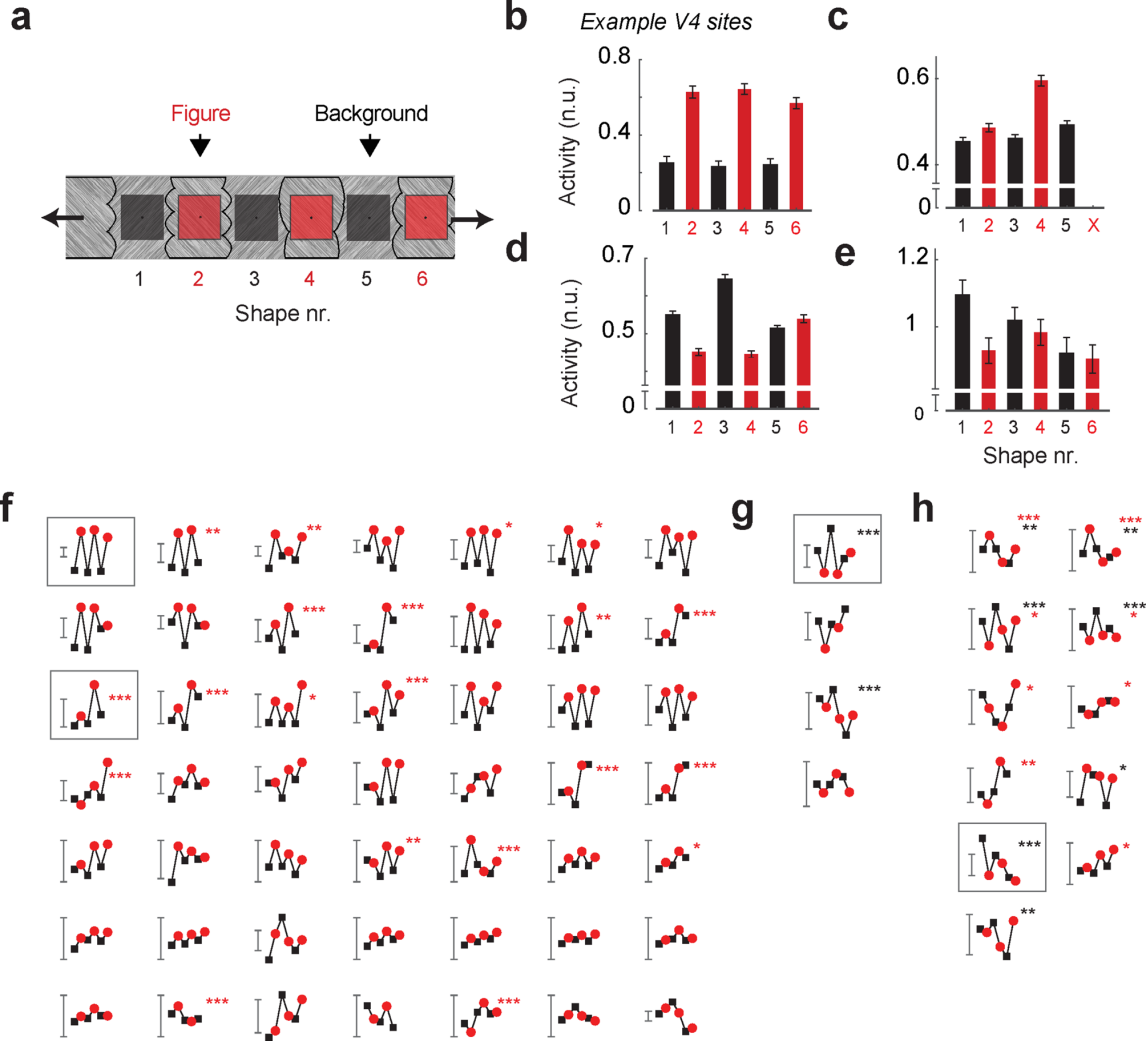
Shape selectivity and FBM in V4. (**a**) Across trials, different figure (red) and background (black) regions were placed in the V4 RFs. The same shapes were present in all experiments so that responses to individual shapes could be compared. (**b**) Example V4 recording site that preferred figures (red) over (black) background regions without distinguishing between the shapes. The bars show the average activity in the time period 50-240ms. n.u. = normalized units, activity normalized to the peak of the transient response. Error bars are +/- 1 s.e.m. (**c**) Example V4 site that preferred figure regions but also preferred one specific figural shape. (**d**) A background preferring site that also showed shape preferences. (**e**) A site with no significant FBM that exhibited significant shape-preference. (**f**) Responses at all significant figure-preferring sites (*N* = 49) sorted by the strength of FBM. The red dots indicate responses to the figure-shapes, the black-dots the background shapes. The scale-bar indicates 0.1 n.u. The asterisks indicate residual significant shape selectivity for figural regions (red stars, ***: *p* < 0.001, **: *p* < 0.01, *: *p* < 0.05, ANOVA). (**g**) Same as d, for background-preferring sites (*N* = 4). (**h**) Same as d, for sites that did not show preferences for figure or background, but exhibited shape selectivity for figure (red stars) or background shapes (black stars) or both.

Previous studies provided evidence that FBM in V1 arises through feedback connections originating in mid-tier visual areas, such as area V4^13,27^. We examined the influence of Gestalt cues on neural activity in V4. The RFs of the neurons at V4 recording sites were 5.9 d.v.a. in size (median FWHM), and many, but not all, V4 RFs overlapped with the boundaries between the image regions, unlike in V1 where RFs fitted entirely within the image regions. We shifted the position of the textured strip across trials, so that different image regions were centered on the V4 neurons’ RFs (Fig. 4a). In this experiment, the spatial relationship of the shapes within the Gestalt strip remained the same, the strip was translated horizontally to different positions to place different figure or background shapes into the aggregate RF of the units. V4 neurons are tuned to shape^34–38^ and shape tuning could therefore influence their responses, in addition to the figure-background organization of the texture strip. We note, however, that convexity, closure and symmetry are shape properties so that tuning to these Gestalt cues reflects a specific form of shape tuning. It is therefore difficult, if not impossible, to dissociate the influence of shape tuning on V4 activity from selectivity to figure or background, especially if the RF size is in the same range as the size of the relevant image regions. However, there are also forms of shape tuning that are orthogonal to figure-background organization. For instance, some V4 neurons might prefer some of the convex shapes over other convex ones.

We focused on the activity that was elicited by the texture strips in the all-cues condition where we expect the strongest influence of figure-background organization. We first determined for each recording site whether it preferred figural or background regions and then compared the shape response within the figure/background category. Figure 4b shows activity at an example V4 recording site that preferred figural image regions 2, 4 and 6 over the background regions 1, 3 and 5 (t-test, t_469_ = 11.7, *p* < 10^− 10^) but responded equally to all figure (ANOVA: F_2,463_ = 0.5, *p* = 0.6) and background shapes (F_2,471_ = 0.34, *p* = 0.7). Figure 4c shows a figure preferring site (t_295_ = 7.9, *p* < 10^− 10^) that also gave a stronger response to one specific figure shape (F_1,294_ = 70, *p* < 10^− 10^). We also recorded from neurons that preferred the background and preferred particular shapes (Fig. 4d) and recording sites at which the neurons were not significantly modulated by figure or background (Fig. 4e). Across the population, 49 of the 75 V4 recording sites preferred figural image regions over background regions (Fig. 4f) and four preferred background regions over figural ones (Fig. 4g). Of the V4 recording sites, 33 distinguished between the figural shapes or between the background shapes (*p* < 0.05, ANOVA, red and black asterisks in Fig. 4f-h) and therefore exhibited a form of shape tuning unrelated to figure-background organization. Of the 33 sites with shape tuning, 22 exhibited FBM and 11 did not (Fig. 4h). Taken together, these results indicate that figural image regions elicit more V4 activity than background regions and that V4 neurons also exhibit tuning to shape features that are unrelated to the Gestalt cues that were tested by us.

**Fig. 5:**
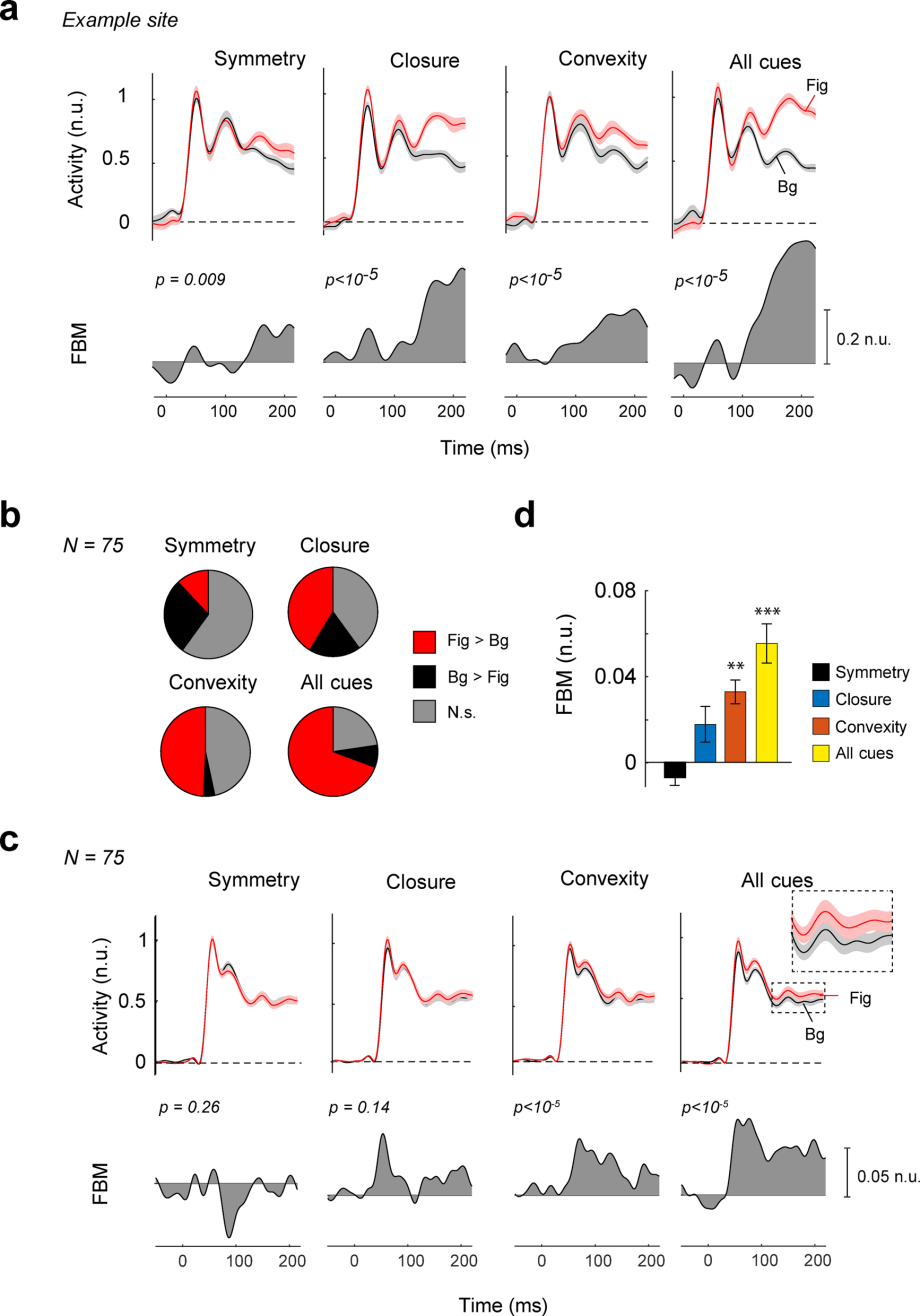
FBM in V4. (**a**) Example V4 recording site. The neuronal responses elicited by figures (red) were stronger than those elicited by backgrounds (black) for each of the Gestalt cues. n.u. = normalized units, activity normalized to the peak of the transient response. The shaded area represents ± 1 s.e.m. across trials. Lower panels, response difference between figural and background regions. (**b**) Proportion of V4 sites with significant (p < 0.05) positive (red) and negative FBM (black). (**c**) Averages time-course of V4 activity for Gestalt-cue defined figures (red) and backgrounds (black). FBM was significant for closure, convexity and when all the cues were combined. (**d**) Average FBM in V4 for the different cues (symmetry = black, convexity = orange, closure = blue, All cues = yellow). Error bars indicate s.e.m. *** = p < 0.001 (Bonferroni corrected), ** = p < 0.01.

We next examined the sensitivity of V4 neurons to symmetry, convexity, closure, and the combination of these Gestalt cues. Figure 5a illustrates the responses of an example V4 recording site that displayed FBM for each Gestalt cue and also if they were combined (t-test, all ps < 0.01). Interestingly, the fraction of sites that preferred figures over backgrounds (Fig. 5b) was larger in V4 than in V1 for all the cues (closure: χ_1_^2^ = 19.6; convexity: χ_1_^2^ = 30.0; combined cues: χ_1_^2^ = 40.0, all ps < 10^− 5^) except symmetry (χ^2^ = 0.28, *p* = 0.6). We also found V4 sites that preferred background regions (black in Fig. 5b) at a proportion that was higher than in V1 (symmetry: χ_1_^2^ = 8.0; closure: χ_1_^2^ = 15.2; all cues: χ_1_^2^ = 11.5, ps < 0.01), except for convexity (χ_1_^2^ = 2.6, *p* = 0.1).

Across the population of V4 recording sites (Fig. 5c), figural regions elicited more activity than background regions when they were defined by convexity (t_74_ = 6.0, *p* < 10^− 5^, Bonferroni corrected), or if the cues were combined (t_74_ = 6.0, *p* < 10^− 5^) and there was a weak trend in the same direction for closure (t_74_ = 2.1, *p* = 0.14). Symmetry did not significantly influence V4 activity (t_74_ = − 1.9, *p* = 0.26) (Fig. 5d). Furthermore, FBM magnitude differed between the cues (weighted linear model, main effect of cue, F_3,222_ = 25.6, *p* < 10^− 5^). Post-hoc tests revealed that FBM was highest in the all-cues condition, weaker for closure and convexity and even weaker for symmetry (all ps < 0.01, Bonferroni corrected). The V4 results were comparable across the three monkeys (Supplementary Figures S4, S5). FBM differed between V4 and V1 (repeated measures mixed ANOVA, interaction of cue and area, F_2.9,521_ = 5.5, *p* = 0.001, Greenhouse-Geisser correction applied) and was stronger in V4 for closure, convexity and if cues were combined (post-hoc independent samples t-tests, all ps < 0.01).

Because the V4 RFs had a similar size as the image regions, we could not dissociate selectivity for figure-background from that for shape. However, a subset of V4 sites (*N* = 19) had smaller RFs (FWHM < 4⁰) that fell entirely within the image regions and did not overlap with the borders. Interestingly, the level of FBM at these V4 sites was significantly larger than at V4 sites with larger RFs (independent samples t-test, t_65_ = 2.5, *p* = 0.02), indicating that part of the FBM is caused by contextual information outside the neurons’ RF (Supplementary Figure S6). The strength of FBM in V4 had a negative correlation with RF size (Pearson’s *r* = − 0.33, *p* = 0.0035), confirming that smaller RFs had stronger FBM than larger RFs.

## The neuronal responses to the Gabor patch and their relation to contrast perception

In the contrast perception task, the monkeys had to make an eye movement to a Gabor stimulus that had a higher contrast than another Gabor stimulus (Fig. 2d, e). One of these Gabor elements (the *test* Gabor) varied in contrast across trials and fell on the ambiguous strip without figure-background structure and the other one, which fell on the strip with Gestalt cues, had a constant contrast (the *reference* Gabor). As was mentioned above, monkey Bo perceived the Gabor elements that were positioned on figures defined by the Gestalt cues as higher in contrast than those that were positioned on the background. In contrast, monkey Da perceived Gabor elements on the background to be of higher contrast. Monkey Du had a strong response bias and his behavioral data could not be used, and we therefore focus on monkeys Bo and Da here. On separate trials we positioned either the reference or test Gabor in the RF of the V1 and V4 recording sites, so that we could examine the activity elicited by these Gabors and how it related to perceived contrast. Did figures increase the neuronal responses elicited by Gabor elements in the monkey that reported an increase in perceived contrast and decrease responses in the other animal?

**Fig. 6:**
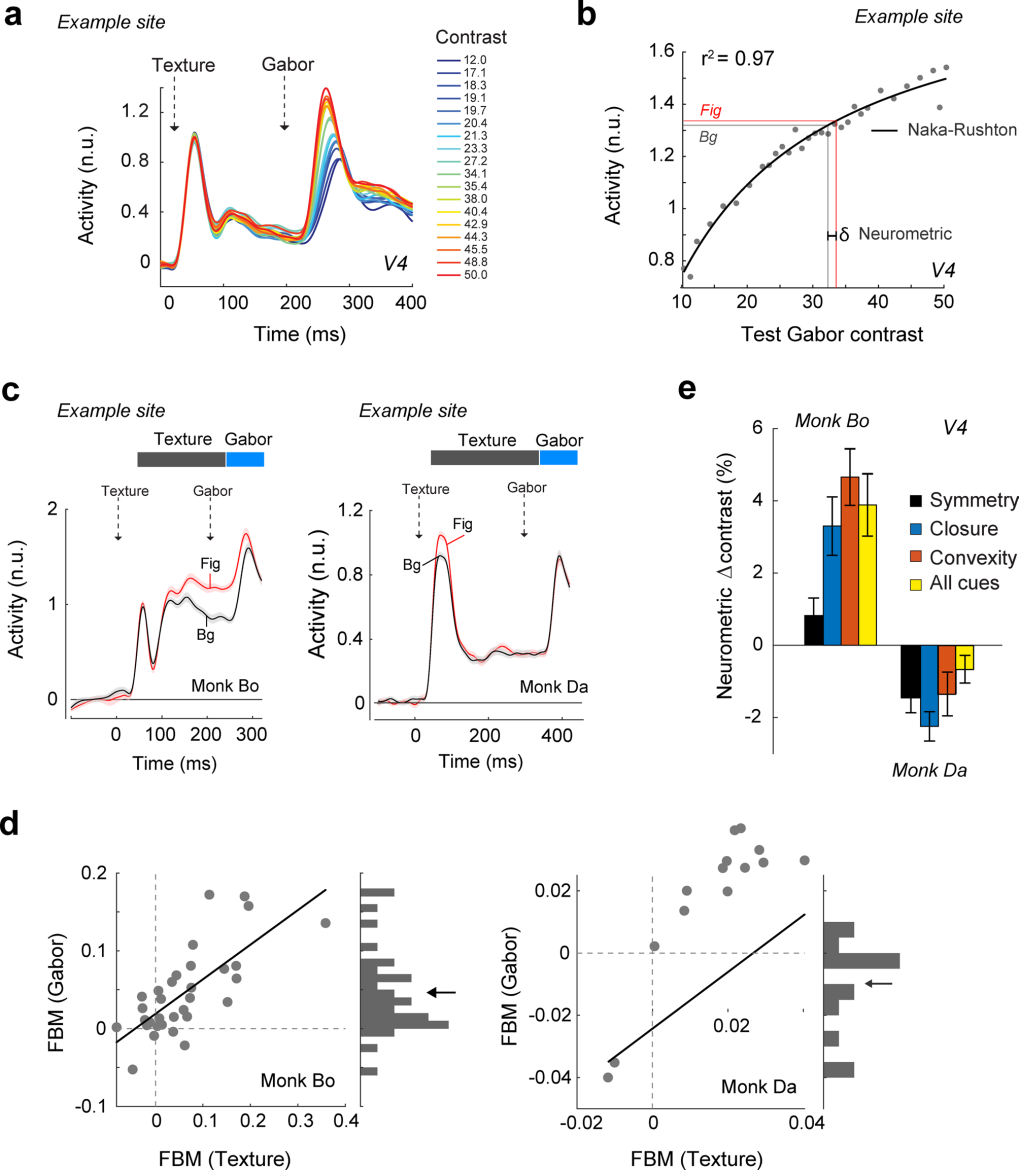
Gabor responses. (**a**) The responses of neurons at an example V4 recording site to test Gabors with different contrast levels. The test Gabor was always shown on the ambiguous texture strip. (**b**) Fit of a Naka-Rushton function to the responses at an example V4 recording site. We inverted the function to predict the PC based on the V4 activity elicited by a reference Gabor on a figure (red line) or background (black line). Reference Gabors always had a contrast of 30%. The difference (δ) can be used to predict the difference in PC between figure and background. (**c**) Activity at example V4 recording in monkeys Bo and Da for figures (red) and backgrounds (black) evoked by the onset of the texture at 0ms and the Gabor at 200ms (monkey Bo) or 300ms (monkey Da). The analysis windows for the texture and Gabor responses are marked in black and blue, respectively. We chose a short time-window after the appearance of the Gabor, excluding time-points after the saccadic eye movement. (**d**) FBM in the Gabor time window was correlated with FBM in the texture window in both animals (Money Bo: *N* = 32 sites, Monkey Da: *N* = 14 sites, both ps < 0.001). The histogram shows the distribution of FBM in the Gabor window in the two monkeys. Arrows indicate the means. (**e**) Predicted difference in PC based on activity difference between Gabors on the figure and background at the V4 recording sites for the different Gestalt cues (symmetry = black, convexity = orange, closure = blue, All cues = yellow). Compare with the behavioral results in Fig. 2e.

We examined responses in a time window following the presentation of the Gabor, before the onset of the eye movement (40-140ms after the appearance of the Gabor). As expected, test Gabor stimuli of higher contrasts elicited stronger transient responses than those with lower contrasts and at a shorter latency (Gawne et al., 1996) in both V1 and V4 (Fig. 6a, Supplementary Fig. S7). Our further analysis focused on area V4, where FBM was stronger than in area V1. The responses to each contrast level were well fit by a Naka-Rushton function (r^2^ > 0.5) at many V4 recording sites (*N* = 62) (Fig. 6b). We next examined the V4 response to the reference Gabor which fell on the figure-background strip and had a constant physical contrast of 30% (Fig. 6c). In monkey Bo, the response to the reference Gabor was stronger if the V4 RF fell on the figure than if it fell on a background region (*N* = 32 sites, paired t-test, t_31_ = 4.7, *p* = 0.00004). In this monkey, the response elicited by the Gabor rode on a sustained response enhancement that was evoked by the figure (Fig. 6c, left panel). In monkey Da, the FBM was most pronounced during the initial transient response and the response to the Gabor rode on slightly weaker activity elicited by the figures (*N* = 14 sites, t_13_= − 2.4, *p* = 0.03) (Fig. 6c, right panel). The additive interaction between FBM and the response elicited by the Gabor was also confirmed when we examined the activity of individual recording sites. In both monkeys, the response difference between Gabors on figures and backgrounds correlated strongly with the level of FBM on a site-by-site basis (monkey Bo: Pearson’s *r* = 0.73, *p* < 10^− 6^, monkey Da: *r* = 0.89, *p* < 10^− 6^, Fig. 6d). In monkey Bo, FBM was generally positive. The level of FBM during the early texture response (black bar in Fig. 6c) predicted the FBM during the later appearance of the Gabor (blue bar in Fig. 6c). In contrast, FBM in Monkey Da was positive shortly after the appearance of the texture strip, but it had become negative during Gabor presentation. Nevertheless, there was a positive correlation between the level of FBM during these two epochs (Fig. 6d). Neurons with weak FBM during texture presentation had a more negative FBM during the Gabor epoch, and neurons with stronger FBM during texture presentation had only weak FBM during the Gabor epoch. We ruled out that these activity differences between monkeys were due to differences in fixation or saccadic behavior (Supplementary Results and Supplementary Figure S8). Furthermore, the activity of V1 neurons elicited by the reference Gabor resembled the V4 response (Supplementary Figure S7).

Does the influence of the Gestalt cues on the amplitude of neural responses to the Gabor element predict the shift in PC? To address this question, we selected recording sites which were well fit by the Naka-Rushton function (r^2^ > 0.5; Fig. 6b). We used the inverse of this function to predict the influence of the change in the V4 response caused by FBM on PC for each recording site (red and black lines and δ in Fig. 6b; see Methods). We took the average of these values as a prediction of the perceived contrast of the Gabor. This analysis predicted that monkey Bo would perceive Gabors that are superimposed on figures with higher contrast than Gabors on backgrounds (Fig. 6e), in accordance with the psychophysical results in this monkey (Fig. 2e). Furthermore, the effects of the different Gestalt cues on neuronal activity were comparable in monkey Bo. In monkey Da, the responses elicited by Gabors on figures were weaker than those on backgrounds (Fig. 6c, d) and the PC predictions were therefore also lower on figures (Fig. 6e), an effect that was again consistent across the various Gestalt cues.

We refrain from drawing strong conclusions about these interindividual differences, which would have required the testing of a much larger sample. Nevertheless, the results do suggest an explanation for the interindividual differences in how figure-ground organization influenced contrast perception. Shortly after stimulus appearance, figural regions generally elicited more activity than background regions, an effect that was consistent across monkeys. However, the Gabor element appeared at a delay and by that time the FBM was maintained in one monkey but had reversed in the other. The activity elicited by the Gabor element rode on top of this activity. As a result, the Gabor elicited more neuronal activity in V4 in the monkey perceiving a higher contrast and less in the monkey perceiving a lower contrast. Hence, the neuronal activity predicted the influence of figure-ground organization on contrast perception.

## Discussion

There is a long history in perceptual psychology about the role of the Gestalt cues in perception. Some of the Gestalt cues determine which image elements group together in perception and others describe what is perceived as figure and what as background^1–3^. We have previously demonstrated that enhanced neuronal activity spreads according to these Gestalt grouping rules^5^. As a result, image elements that are similar, move coherently or in each other’s good continuation are co-selected by attention. Here we focused on the Gestalt cues of closure, convexity and symmetry, which influence figure-background perception, and we measured their influence on neuronal activity in areas V1 and V4. Related previous work focused on size, one of the Gestalt rules that influences figure-background perception. Image regions that are small are more likely to be perceived as figural. These figural elements increase perceived contrast^31^ and enhance responses in the visual cortex^10,11^. Here, we chose to present figure regions that were irrelevant for behavior, that had the same size as the background and were displayed across the full width of the screen to avoid pop-out effects. We found that convexity and closure influence figure-background modulation for surfaces, even if they are equal in size and irrelevant for behavior.

### The influence of Gestalt cues on figure-background organization and contrast perception

In agreement with previous work^2,3,7,39,40^, we found that convex and closed image regions were more likely to be perceived as figures than concave and open image regions, in particular when cues were combined. Symmetry was not effective as a grouping cue in this experiment. This is somewhat surprising as, although symmetry has been considered to be a weak cue in some studies^7^, other studies have identified it as an important grouping cue^8,9,41,42^. The weaker influence of symmetry in our stimuli may be related to the use of vertically displaced Gestalt strips, as symmetry has been shown to be most effective as a grouping cue in central vision, i.e. when the figure is within 3 d.v.a of fixation^41,43^.

On average, observers perceive Gabor elements placed on a figure to be of higher contrast than those placed the background^31^. Here we used contrast perception as an indirect measure for figure-background organization, because this approach avoids the shifts of attention that occur when subjects would have to report about figure-background organization. Most of our human subjects perceived the contrast of Gabor elements on figures to be higher than those on backgrounds, especially when figures were defined by multiple cues. However, there was inter-individual variation, and a few participants perceived Gabor elements on the figure to be of lower contrast than those on the background. We were able to train two of three monkeys to reliably report perceived contrast. Their results also exhibited interindividual variability, because one monkey perceived the Gabor elements on figures to be of higher contrast and the other animal exhibited the opposite effect. Interestingly, figures elicited more activity than the background in V1 and V4 of both animals, but there were differences in how the FBM interacted with the representation of the Gabor elements. Specifically, figures boosted the representation of the Gabor elements in monkey Bo, for which perceived contrast was higher, during a late phase of the V1 and V4 responses. In monkey Da, figures rather reduced perceived contrast. In this monkey, FBM was more transient and by the time of test Gabors presentation it even reversed in sign so that that figures weakened their representation. This transient FBM pattern differs from previous studies in which monkeys reported about figure-ground perception because FBM was stronger and sustained^24,44^. It is therefore conceivable that tasks in which figural-ground assignment is relevant for the task would also have changed monkey Da’s FBM into a more sustained pattern. Taken together, these interindividual differences imply that perceived contrast can be used as an indirect measure for figure-background structure across larger groups of subjects, but also that it is an unreliable marker for the figure-background perception of individual participants.

### The influence of Gestalt cues on neuronal activity in areas V1 and V4

FBM occurred in area V1 and V4 when figures were convex and closed, and in particular when the cues were combined, but symmetry did not produce FBM. Hence, the relative strengths of the influences of these Gestalt cues on neuronal activity were reminiscent of their effects on contrast perception. Although the contrast judgement tasks used here diverted attention from the textures, FBM was present, especially when all cues were combined. This is of interest, because some previous studies did not observe FBM when attention was directed elsewhere^45–47^. However, it is possible that figural regions, especially the all-cues condition, were salient and attracted bottom-up attention.

The Gestalt cues of convexity, closure and symmetry are properties of the shapes of everyday objects. They might therefore represent statistical biases that are useful in the assignment of image regions to figures or background^4^. Indeed, images regions that are part of familiar shapes, such as faces or fruits, have a figural status and they are processed with priority over the background^8,32,48^, implying a link between the neuronal mechanisms for object recognition and figure-background organization^49^. In this view, convexity, closure, and symmetry represent shape properties that are common to a large set of everyday objects and are expected to be analyzed in the brain regions that contribute to object recognition.

Many of our V4 recording sites had RFs that included the borders of the image regions, and we could therefore not fully dissociate tuning to visual shape from the tuning of the Gestalt cues. We also observed shape tuning in V4 that was unrelated to the processing of Gestalt cues by comparing the responses between figural regions and between background regions, which is in accordance with previous work^25,38,50^. However, at the population level V4 neurons had stronger responses to figural regions than to background regions, especially if all Gestalt cues were combined. Interestingly, these increased responses occurred in a task in which the monkeys could ignore figure-background segmentation cues and this supports previous psychophysical results in humans showing that figural regions are more salient than background regions^48,51,52^. Nevertheless, some V4 neurons exhibited a preference for background regions (Fig. 5b) and were tuned to e.g. concave regions^37,38,53,54^. It is conceivable that these cells represent competing perceptual hypotheses to assign a figure-status to the concave image region. In accordance with the perceptual prominence of the figural regions, FBM was also pronounced in a subset of V4 recording sites with smaller RFs that fell within the image regions. We note, however, that we estimated the RF-size as the full-width-at-half-maximum of the visual field regions from which we could elicit responses with small stimuli (FWHM, see Methods) and the borders presumably fell in less excitable RF regions of these recording sites. The situation was different for our V1 recordings because the RFs were smaller and did not overlap with the borders between image regions. In V1 FBM was therefore a contextual effect driven by information from outside the RF. Future studies could test whether V1 neurons would also exhibit preferences for Gestalt-related shape properties if the shape features fall in their RFs, because many of them exhibit selectivity that goes beyond simple orientation tuning^55,56^.

Models of figure-background assignment^49,57^ hold that feedback from higher visual areas is a likely source of FBM in V1, although researchers have also proposed important roles for feedforward connections from lower areas^58^ and horizontal connections that interconnect neurons with different RFs in the same areas (e.g.^59^). However, it seems unlikely that models without feedback connections can explain FBM in V1 if foreground regions have the same size as background regions and figure-background organization depends on convexity and closure.

Our results might indicate that higher cortical areas, including area V4, detect the shape of foreground objects and feed back to lower cortical areas. FBM in V1 was weaker than in area V4, as has also been observed in studies that used small figures on larger backgrounds^10–12,23,24,33,60–62^. In accordance with a role for feedback, a recent study in mice demonstrated that optogenetic silencing of higher cortical areas suppresses FBM in area V1^13^.

An important advantage of FBM in V1 is that it provides a high-resolution representation precisely delineating where a foreground object ends, and the background begins. These segmentation signals are useful, for example, when grasping an object, because the fingers need to be placed precisely on edges that are part of the same object^63^. The V1 segmentation signals can then be read out by higher cortical areas to guide behavior^64^. Future studies could examine how the segmentation processes that follow Gestalt rules generalize to natural scenes with objects that can be manipulated, to increase our understanding of how lower and higher visual areas work together to extract behaviorally useful coherent object representations.

## Methods

### Stimuli

#### Figure-background and ambiguous strips

We created textured strips with regions with an alternating orientation (Fig. 1c). In some strips the figure-background organization was ambiguous and they served as a baseline condition. In other strips, figural regions were defined by the Gestalt cues: symmetry, closure, convexity, or a combination of these three cues (all-cues condition) (Fig. 1c). Each strip was 7 degree of visual angle (d.v.a.) in height and extended across the full width of the screen (38.7 visual degrees). The strips were placed on an equiluminant gray background. Each strip contained of six regions, three figure and three background regions. We chose to use multiple-region displays as previous research has shown that in two-region displays, cues such as convexity and symmetry have weak effects whereas in multiple-region displays regions the figureness of these cues was enhanced^41,65–67^. Each region within the strip had an average width of 5 visual degrees. Regions were separated by black lines (width: 0.2 degrees). All regions consisted of textures with white, oriented line elements (thickness: 0.04 degrees, length: 0.8 degrees, orientation: 45 or 135 degrees) on a dark grey background. The texture orientation of adjacent image regions always differed by 90 degrees.

### Experiment 1: Influence of Gestalt cues on figure-background perception in human participants

#### Participants

Ten healthy participants participated in the experiment (M = 24.7 years, SD = 1.9 years). They had normal or corrected-to-normal vision. Participants provided informed consent and received financial compensation for their time. The experiments were approved by the ethical committee of the University of Amsterdam, the Netherlands. All experiments were performed in accordance with relevant guidelines and regulations and were in accordance with the Declaration of Helsinki.

### Procedure and analysis

Participants were seated in a dark room. Stimuli appeared on a grey (luminance 46.3 cd/m²) computer screen (Windows controlled Dell computer, 19 inch screen) at 1024 × 768 resolution with an 85 Hz refresh rate at a 57 centimeter viewing distance. The viewing distance was controlled by a chin rest. Participants had to fixate on the fixation point in the middle of the screen during the entire trial. Eye positions were evaluated online using an eye tracker (Eyelink CL, SR Research Ltd, Mississauga, Ontario, Canada) with a sampling rate of 1000 Hz using the Eyelink Toolbox for MATLAB (MATLAB, The MathWorks Inc, Natick, Massachusetts, United States). During the experiment, a trial was aborted when the participants’ gaze deviated from the fixation point by more than 1 d.v.a. Aborted trials were repeated later in the session. Participants were asked to respond as rapidly as possible while keeping the number of errors to a minimum. They were kept naïve with respect to the aim of the experiment.

A trial was initiated when the subject fixated on a red fixation point for 200ms (diameter: 0.5 degrees) on a grey background. A strip (Fig. 1b) was presented for 400ms, with the center of the strip 6.23 degrees below the fixation point. Participants were asked to indicate whether the region directly below the fixation point was a ‘figure’ or a ‘background’ region by pressing a key (↑ for figure, ↓ for background). Participants could take as much time as needed to respond and they did not receive feedback about their performance. We used a pseudo-random blocking design, in which the five conditions were presented in a random order with 32 trials per strip per block. The shapes within the strips were shifted horizontally so that a figure was cued on half the trials, and a background in the other half. There were 4 blocks in total, resulting in 640 trials per subject. The strength of the Gestalt cues on figure-background perception was estimated as the percentage of times the participants responded ‘figure’ when we presented a figure, and ‘background’ when we presented a background at the cued location. Cues that induce a clear figure-background percept would therefore have a figureness score of 100%, whereas cues that do not induce any figure-background percept would have a figureness score of 50% (chance level). We assessed the significance of figureness with right-sided Wilcoxon signed rank tests.

### Experiment 2: The influence of Gestalt cues on perceived contrast in human participants

#### Participants

Ten healthy participants participated in the experiment (M = 25.9 years, SD = 9.2 years), five of these participants also participated in Experiment 1 (Experiment 2 was run first in these participants). They had normal or corrected-to-normal vision. Participants provided informed consent and participated in exchange for a monetary reward. The experiments were approved by the ethical committee of the University of Amsterdam, the Netherlands. All experiments were performed in accordance with relevant guidelines and regulations and were in accordance with the Declaration of Helsinki.

### Procedure

The setup and eye tracking system were the same as in experiment 1. Figure 2a shows an overview of the trial structure of experiment 2. Participants performed a contrast discrimination task with a temporal two-alternative forced-choice design. They initiated a trial by directing their gaze for 200ms to a red fixation point (diameter: 0.5 degrees) on a grey background. The trial always started with the presentation of the ambiguous strip for 100ms before a reference Gabor element with a Michelson contrast of 30% was superimposed for another 300ms. After a short interval (170ms), we presented a figure-background strip for 100ms and then a superimposed test Gabor element for another 300ms, which varied in contrast across trials. Afterwards, the strip disappeared and the fixation point turned green, cueing the subject to respond by pressing a keyboard arrow (↑ if the first Gabor element was higher in contrast, ↓ if the second Gabor had higher contrast).

The Gabor patches were vertical and generated by multiplying a sine wave of 3 cycles/degree with a Gaussian kernel with a standard deviation of 0.33 degrees, within an aperture of 2 degrees. We embedded a Gabor element in the textured surface by adding it to the luminance of the underlying texture^31^. The average luminance of the Gabor elements was 30 cd/m². The Gabor was always placed in the middle of the strip, 6.23 visual degrees below fixation. The orientation of the texture of the region on which the Gabor was presented was always 135 degrees.

We used a 2-down/1-up staircase procedure to measure the PC of the test Gabor. The contrast difference between the two Gabor elements was decreased upon two consecutive correct responses and increased after one error. There were independent staircases for the four types of Gestalt cue-defined figures and for the figure and background conditions. Two staircases were used for each condition, one starting with a lower contrast for the reference Gabor, and the other with a higher contrast. This resulted in in 16 staircases in total, which were presented in an interleaved, pseudorandom order. To maintain the participants’ motivation, we also included 10% randomly interleaved ‘easy’ trials, in which the test contrast was either 22 or 38% and thus differed substantially from the contrast of the reference Gabor (30% contrast). We restricted the contrast of the test Gabor to 22–38% contrast and used a step size of 2% until the first reversal, 1% until the second reversal and 0.5% for later trials. The 2-down/1-up staircase converged to an accuracy of 75% correct, which we refer to as the Contrast Discrimination Threshold (CDT), and we derived PC from this measure (see below). Each staircase had 30 trials and was repeated 3 times per session to obtain multiple estimates of the PC. Feedback was provided on error trials, but only if the contrast difference between the reference Gabor and the test Gabor was larger than 4%. Participants performed 512 trials per session (in 20–30 min) and a total of six sessions. Staircases were reset between sessions.

### Analysis

The CDT was defined as the contrast level necessary to produce 75% correct performance and was estimated as the average of the contrast values on the last 3 trials per session. We calculated the CDT on both ascending (CDT_asc_) and descending staircases (CDT_des_) and used these values to estimate the change in perceived contrast of the test Gabor relative to the reference Gabor (PC). According to Weber’s law, performance is better on ascending stairs than descending stairs (as the average contrast of the two Gabors is lower on ascending staircases) and therefore we computed the geometric mean, which takes Weber’s law into account when estimating PC (for details, see^31^):

1 $$\:\varDelta\:PC\:=30-\sqrt{{CDT}_{asc}{CDT}_{des}}$$

Where 30 was the percentage contrast of the reference Gabor. Aborted trials and the 10% of easy trials were not included in the analysis. We determined PC for each Gestalt cue per session for each subject. We used two-sample t-tests to determine significance of the PC difference between Gabors situated on figures and backgrounds.

### Experiment 3: Neuronal and behavioral responses to Gestalt cues in macaque monkeys

#### Participants

Three male rhesus macaques (*Macaca mulatta*) participated in this experiment (monkey Bo: 8 kg, monkey Du: 7.5 kg, monkey Da: 7.5 kg). All procedures complied with the NIH Guide for Care and Use of Laboratory Animals and were in accordance with the ARRIVE guidelines, and were approved by the Institutional Animal Care and Use Committee of the Royal Netherlands Academy of Arts and Sciences. Monkeys were socially housed in pairs. In previous studies, monkey Bo had been trained on visual attention tasks, monkey Du had been trained on curve tracing and working memory tasks and monkey Da had been trained on curve tracing and on tasks in which reward expectancy was varied.

#### Surgeries

We implanted a titanium head-post (Crist instruments) for head-stabilization under aseptic conditions and general anesthesia. Anesthesia was induced with ketamine (10 mg/kg i.m.) and xylazine (0.6 mg/kg i.m.). In cases where sedation was not deep enough, we also supplemented dormicum (1–2 mg/kg i.v.). The monkey was then intubated and infused with Ringer’s lactate (10–15 mL/kg per h). Anesthesia was maintained after intubation by ventilating with a mixture of air and O2 in 2:1 ratio, supplemented with 0.8–1% isoflurane and fentanyl (0.005 mg/kg i.v.). After the operation, the monkeys received analgesics (Temgesic; 0.005 mg/kg i.m.) and antibiotics. After recovery from the operation, the monkeys were trained until they reached stable performance on the figure–ground task (details in^68^. Monkeys then underwent a second surgery under general anesthesia in which eight Utay arrays of 4 × 5 or 5 × 5 micro-electrodes (Blackrock Microsystems) were chronically implanted in area V1 and V4 in the left (Monkey Bo, Da) or right hemisphere (Monkey Du) (details in^26,69^).

### Procedure and task

The monkeys were seated in a primate chair with their heads fixed. Stimuli appeared on a 19-inch Dell Triniton linearized CRT monitor (1024 × 768 resolution with an 85 Hz refresh rate), at a viewing distance of 52 cm. We measured the position of the pupil of the right eye with a Thomas Recording eye tracker system with an infrared camera (frame rate: 230 Hz). A tube connected to a juice system (Crist Instrument Co., Inc) provided liquid rewards. The monkeys performed a spatial two-alternative forced choice contrast discrimination task in which two strips were presented simultaneously (Fig. 2d). The trial started when the monkeys directed their gaze to a 1.1 degree diameter fixation window centered on a red fixation dot (diameter: 0.5 degrees) in the middle of a grey (luminance: 38.1 cd/m^2^) screen for 200ms. Then the strips were shown and after a delay (200ms for monkey Bo and Du or 300ms for monkey Da), two Gabor elements appeared, one per strip. The task was to make an eye movement to the Gabor element with the highest contrast. For monkeys Bo and Du the fixation dot turned green when the Gabor elements appeared, cuing them to make the eye movement within 2000ms. For monkey Da, the fixation point turned green 200ms after the appearance of the Gabor elements, which allowed us to record more neuronal data before the onset of the eye movement. The size of the target window was between 2 and 6 d.v.a. in radius, scaling with the eccentricity of the target. When the monkeys broke fixation prematurely, the trial was aborted.

We trained the monkeys in several stages on this relatively difficult task. They were first trained to direct gaze to a fixation point and to make eye movements towards visual stimuli. Subsequently, they were trained to make eye movements to a single high contrast Gabor presented on a gray screen. We then introduced a second Gabor of lower contrast on the opposite side of the fixation point, and the monkeys had to make an eye movement towards the Gabor of the highest contrast. We later added the texture strips and temporarily added a pink cue to the Gabor with highest contrast, given the reduced visibility of the Gabors. Once accuracy recovered, we removed the pink cue and added the Gestalt cues to the strips so that the Gabors could be placed on figural or background regions. These strips were never task relevant.

#### Figure-background stimuli

The figure-background strip and the ambiguous strip appeared at the same time, one above and below the fixation point (‘upper’ and ‘lower’ strip, respectively). The upper and lower positions of the figure-background and ambiguous strip were randomly assigned. We placed the two strips at a similar vertical distance from fixation (range 1.1–10 degrees) and ensured that for a given trial, either the center of a V1 or a V4 RF fell in the center of a region of the lower strip (see ‘Electrophysiology’ below and Supplementary Figure S1). Each strip contained six regions, three figure and three background regions. Across trials, we moved the strip horizontally to ensure that across trials the three different figure and background regions fell in the RF (for RFs with an eccentricity > 6 degrees only two figure regions fell in the RF). The distance between the center of the aggregate RF (i.e. the mean x and y position of all RFs of recording sites on the same array) and the closest region boundary was at least 2 degrees. Figure 2d illustrates how we avoided the vertical alignment of the regions of the two strips, which otherwise might have influenced the figural status of ambiguous region^70^. We placed the reference Gabor (30% Michelson contrast) on the figure-background strip and the test Gabor on the ambiguous strip. Both Gabor elements were placed in the center of an image region. The horizontal offset of the two strips caused an equivalent horizontal offset between the Gabor elements (Fig. 2d).

#### Staircase procedure

We intermingled ascending and descending staircases to determine PC of the test Gabor and we also used independent staircases for different RF positions. We used the Quest algorithm for monkeys Bo and Du^71^. This method chooses a contrast value (between 10 and 50% contrast) for each trial that is the Bayesian estimate of the contrast discrimination threshold (75% correct). To ensure the monkeys stayed motivated, we also added 15% ‘easy’ trials, in which the contrast of the Gabor was either 10% or 50%. Monkey Du developed a strong bias to select the lowermost Gabor, particularly when the figure-background strip was in the lower position. This bias strongly affected estimates of PC for this monkey and we therefore could not further analyze his behavioral performance. For this reason, we switched to using the method of constant stimuli in monkey Da as we considered this procedure to be less likely to cause response biases. Here, test Gabor contrasts were randomly selected from a constant set of contrast values ranging from 10 to 60% contrast. We presented the trials in blocks of 100 trials per Gestalt cue and the order of these blocks was pseudo-randomized. Trials in which fixation was lost were repeated at the end of the block.

#### Perceived contrast

To compute psychometric functions, we binned the contrasts into 29 bins, ranging from 0 to 60% Michelson contrast. We fit the number of correct trials at each contrast bin $\:\psi\:\:$using a logistic function using a maximum likelihood criterion (PAL_PFML_FitMultiple of the Palamedes toolbox^72^):

2 $$\:\psi\:\left(x;\alpha\:,\beta\:,\gamma\:,\lambda\:\right)=\:\gamma\:+\:\frac{1-\gamma\:-\lambda\:}{1+\mathrm{e}\mathrm{x}\mathrm{p}(-\beta\:\left(x-\alpha\:\right))}\:$$

The free parameters were the slope $\:\beta\:$, threshold $\:\alpha\:$, guess-rate $\:\gamma\:\:$and lapse-rate $\:\lambda\:$ of the function. The guess-rate (i.e. the number of incorrect responses at zero contrast) and lapse-rate (i.e. the number of incorrect responses at theoretical infinite contrast) were constrained to be the same for all conditions. Similarly, the slope of the function was constrained to be the same for all conditions, ensuring only parallel shifts of the psychometric function relative to the x-axis. The guess- and lapse rate were constrained to lie between 0 and 0.4. PC of the reference Gabor was taken as the threshold parameter α and the difference in PC between the conditions was taken as $\:{{\upalpha\:}}_{fig}-{{\upalpha\:}}_{bg}.$ Significance was assessed using bootstrapping. We derived null distributions by randomly drawing an identical number of trials at each contrast level as in the original dataset. Under the null hypothesis, the probability of a correct response is the same for figure and background trials, we therefore drew performance scores from a binomial distribution with a probability of success that was the average probability of a correct response across figure and background trials. After drawing surrogate trials for all contrast levels, figure-background conditions and cues, we refit the data using the same approach as described above and we calculated the difference in PC between figure and background for the surrogate data. We repeated this procedure 1000 times to derive a null distribution of perceived contrast differences expected by chance. The significance was assessed by a two-tailed procedure as the proportion of unsigned perceived contrast differences from the null distribution which were greater than or equal to the experimental values.

#### Electrophysiology

Neural activity in V1 and V4 was recorded with a TDT multi-channel acquisition system (Tucker-Davis Technologies, Alachua, FL). Electrophysiological signals were digitized at 24.4 kHz and band-pass filtered (500–5000 Hz) to isolate high-frequency spiking activity. This signal was full-wave rectified (negative becomes positive), low-pass filtered (200 Hz) and resampled at a rate of 763 Hz to obtain the multi-unit activity (MUA). MUA reflects the spiking activity of neurons within ~ 100–150 μm of the electrode and this activity is similar to that obtained by pooling across single units^68,69,73,74^. A notch filter was applied to the MUA signal in order to remove line noise (50 Hz) before low-pass filtering the data with a 5th order Butterworth filter with a corner frequency at 40 Hz.

### Receptive field mapping

We mapped the RFs of V1 recording sites with a drifting light bar (width: 0.25 degrees, length: 19.8 degrees, luminance: 74 cd.m^− 2^) that moved in one of the four cardinal directions (speed: 15.75 degrees/s) and elicited a robust response at the V1 recording sites. We fit a Gaussian function to the neuronal response to each bar direction and estimated the onset and offset of the response as the 2.5th and 97.5th percentile of the Gaussian function and calculated the RF borders as reported previously^69^. We also determined the average signal-to-noise ratio during the RF mapping session (SNR_RF_) by dividing the peak of the Gaussian by the standard deviation of the baseline response across trials (time window − 100-0 ms relative to stimulus onset). We discarded recording sites with SNR_RF_<2 in the RF mapping task. The average V1 RF size, taken as the square-root of the area, was 2.4 degrees (range 0.35 to 6.6 degrees) and the mean eccentricity of the RFs was 3.8 degrees (range 0.35 to 8.3 degrees).

We mapped V4 RFs with luminance-defined squares (size: 1 × 1 degree) flashed on a black background in a 21 × 21 degrees square grid in the lower visual field (right visual field for Monkey Bo and Da, left for Du). On each trial, the monkeys had to fixate for 500ms within a 1.1 degrees fixation window centered on a red dot (diameter 0.5 degrees) in the middle of a black screen (luminance 2.45 cd.m^− 2^). Per trial, two white squares (luminance 73.75 cd.m^− 2^) were presented for 150ms with a 100ms interval between them. For each recording site, we took the average response, R_*xy*_, to each square (with coordinates x and y) in a time-window from 0-150ms. To estimate the location and size of the RF, we fit a 2D Gaussian to the responses. Only well-fit recording sites with an r²>0.4 were included in the dataset. The median V4 full-width-at-half-maximum (FWHM) RF size, defined as 2.35 times the standard deviation of the Gaussian, was 5.9 d.v.a. and the median eccentricity was 5.4 d.v.a. (range 1.2 to 9.7 d.v.a.).

All V1 and V4 RFs were in the lower visual field (Supplementary Figure S1). RF sizes and locations of recording sites on the same array were averaged to compute an aggregate RF, which was used to determine the position of the Gestalt strips (Supplementary Figure S1).

### Data analysis

We first calculated the mean signal across all trials for a given recording site and then smoothed this average with a 20-sample locally-weighted smoothing function (Matlab ‘smooth.m’ function using the ‘lowess’ option). We then baseline-corrected each trial using a smoothed estimate of the baseline across trials to remove drifts in the baseline. The estimate was calculated using a robust locally-estimated scatterplot smoothing function using 30% of the data in each window (Matlab ‘smooth.m’ function using the ‘rloess’ option). The data were normalized by dividing by an estimate of the peak visual response of the recording site to strips that were well-centered on the RF. The peak response was estimated by smoothing the average MUA response across all well-centered trials with a 20-sample moving-average window and then taking the maximum in the time-period 30-100ms. Hence, activity is shown in normalized units where a value of e.g. 0.1 indicates 10% of the peak response. The data was recorded across several days (monkey Bo: 13 days, monkey Du: 17 days, monkey Da: 18 days) and individual recording sites occasionally picked up noise in some of the recording session (e.g. due to a poor connection to the amplifier). We excluded these sessions using an SNR-based criterion. We calculated the SNR for each site per day by dividing the peak response (as estimated above) by the standard deviation of baseline activity (averaged in a window from − 100 to 0ms) across trials. Recording days with an SNR < 0.5 were excluded on a site-by-site basis. We occasionally observed a recording site with an unusual response on a single day. We detected outliers by comparing the responses of a recording site per session elicited by strips well-centered on the RF to the average response across sessions by calculating the Pearson correlation. Only days with a Pearson correlation value > 0.85 were included. We included recording sites with an overall SNR > 1 (unlike RF mapping where the SNR criterion differed) and an RF which was well-centered on the image regions. We excluded sites for which the RF center was more than 1 d.v.a. from the center of the regions in the x-direction and more than 2 d.v.a. in the y-direction. These criteria resulted in the inclusion of 111 sites in V1 (19 in Monkey Bo, 67 in monkey Du and 25 in monkey Da) and 75 in V4 (32 in monkey Bo, 29 in monkey Du and 14 in monkey Da).

FBM was considered to be significant based on an independent samples t-test that compared trials in the figure and background condition (time window of 100-240ms for monkeys Bo and Du and 100-340ms for monkey Da as the Gabors appeared at 300ms in this monkey compared to 200ms in the other two). For V4 data, we used time windows that started at 50ms, because FBM in V4 starts earlier than in V1^24^. To identify recording sites at which figure responses were higher than background responses and vice versa we applied two single-tailed tests. At the population level, we performed separate weighted paired t-tests to assess the difference between figure and background activity in response to the different cues. We weighted the data by the inverse of the number of recording sites contributed by each monkey so that each animal contributed equally to the population average.

### Shape selectivity

The three figure and background regions of each strip had a different shape and we tested whether V4 recording sites were tuned to these shapes in addition to their preference for figure or background. To assess the significance of shape selectivity we used ANOVAs on figure- and background shapes separately to isolate shape-selectivity from FBM with a statistical threshold of *p* < 0.05.

### Neuronal responses to the Gabor elements

To examine responses evoked by the appearance of the Gabor stimulus we averaged activity in a time window from 40-140ms after its appearance. We first identified recording sites with clear contrast response functions to the test Gabor with varying contrast. For monkey Bo, we grouped the contrasts of the staircase into 20 bins with a similar number of trials. For monkey Da, we used the 31 fixed contrast values and binning was not required. We fitted a Naka-Rushton equation to the contrast response function:

3 $$\:y\left(x\right)=b+\frac{a{x}^{n}}{{x}^{n}+{c}_{50}^{n}}$$

Where *y* is the response for a given contrast level/bin, *x* is the contrast which ranges between 0 and 1. *b* is the response level at zero contrast, *a* is a gain parameter controlling the saturation of the curve at high contrasts, c_50_ is the contrast sensitivity controlling the position of the curve along the x-axis and *n* controls the steepness of the function. We included recording sites where the fitted curve explained more than 50% of the variance in responses (r^2^ > 0.5) for further analysis. We inverted the Naka-Rushton equation for each recording site to predict the difference in PC of Gabors on figures and backgrounds as the mean value across recording-sites (Fig. 6b):

4 $$\:x\left(y\right)=\:{\left(\frac{{yc}_{50}^{n}-b{c}_{50}^{n}}{a-y+b}\right)}^{1/n}$$

## Supplementary Information

Below is the link to the electronic supplementary material.

Supplementary Material 1

## Data Availability

All data and analysis scripts are publicly available: https://doi.org/10.17605/OSF.IO/T5RCU.
