# Supplementary material for: Gestalt laws enhance the representation of figures over backgrounds in the visual cortex and influence contrast perception
Source: Sci Rep. 2026 Apr 7;16:11685. doi: 10.1038/s41598-026-45730-8 (PMC13061894; doi:10.1038/s41598-026-45730-8)
Supplement: Supplementary file 1 — Supplementary Material 1 [file 41598_2026_45730_MOESM1_ESM.pdf]

Supplementary information

Gestalt laws enhance the representation of figures over backgrounds in the visual cortex and influence contrast perception

Anne F. van Ham[1#], Danique Jeurissen[1,2#], Matthew W. Self[1,3^], Pieter R. Roelfsema[1,4,5,6^*]

[1]Department of Vision & Cognition, Netherlands Institute for Neuroscience, Meibergdreef 47, 1105 BA, Amsterdam, the Netherlands.

[2] Center for Neural Science, New York University, 4 Washington Pl, New York, NY, USA.

[3] School of Psychology and Neuroscience, University of Glasgow, Glasgow, Scotland.

[4]Department of Integrative Neurophysiology, Center for Neurogenomics and Cognitive Research, VU University, De Boelelaan 1085, 1081HV Amsterdam, The Netherlands.

[5]Department of Neurosurgery, Academic Medical Center, Postbus 22660, 1100DD Amsterdam, The Netherlands.

[6]Laboratory of Visual Brain Therapy, Sorbonne Université, Institut National de la Santé et de la Recherche Médicale, Centre National de la Recherche Scientifique, Institut de la Vision, Paris F-75012, France.

[#]/^These authors contributed equally. *Corresponding author: p.roelfsema@nin.knaw.nl.

No. of Figures: 6; No. of Supplementary Figures: 8;

Analysis of variation of eye position and (micro-) saccades around the fixation point

Fixation behavior was very accurate for all three monkeys (Supplementary Figure S8a). There were differences in micro-saccade rate between the monkeys, because monkey Da made microsaccades on 20% of the trials, whereas Bo and Du made microsaccades on 6% of the trials. Furthermore, monkey Da's microsaccades were smaller and they tended to be in the vertical direction (Supplementary Figure S8b,c). We analyzed trials without micro-saccades and observed that the magnitude of FBM was very similar to FBM analyzed across all trials, and the statistical results of our analysis were unaffected. In all monkeys, the saccadic landing points in the main task were accurate with a slight decrease in accuracy at higher target eccentricities (Supplementary Figure S8d).

Supplementary figures

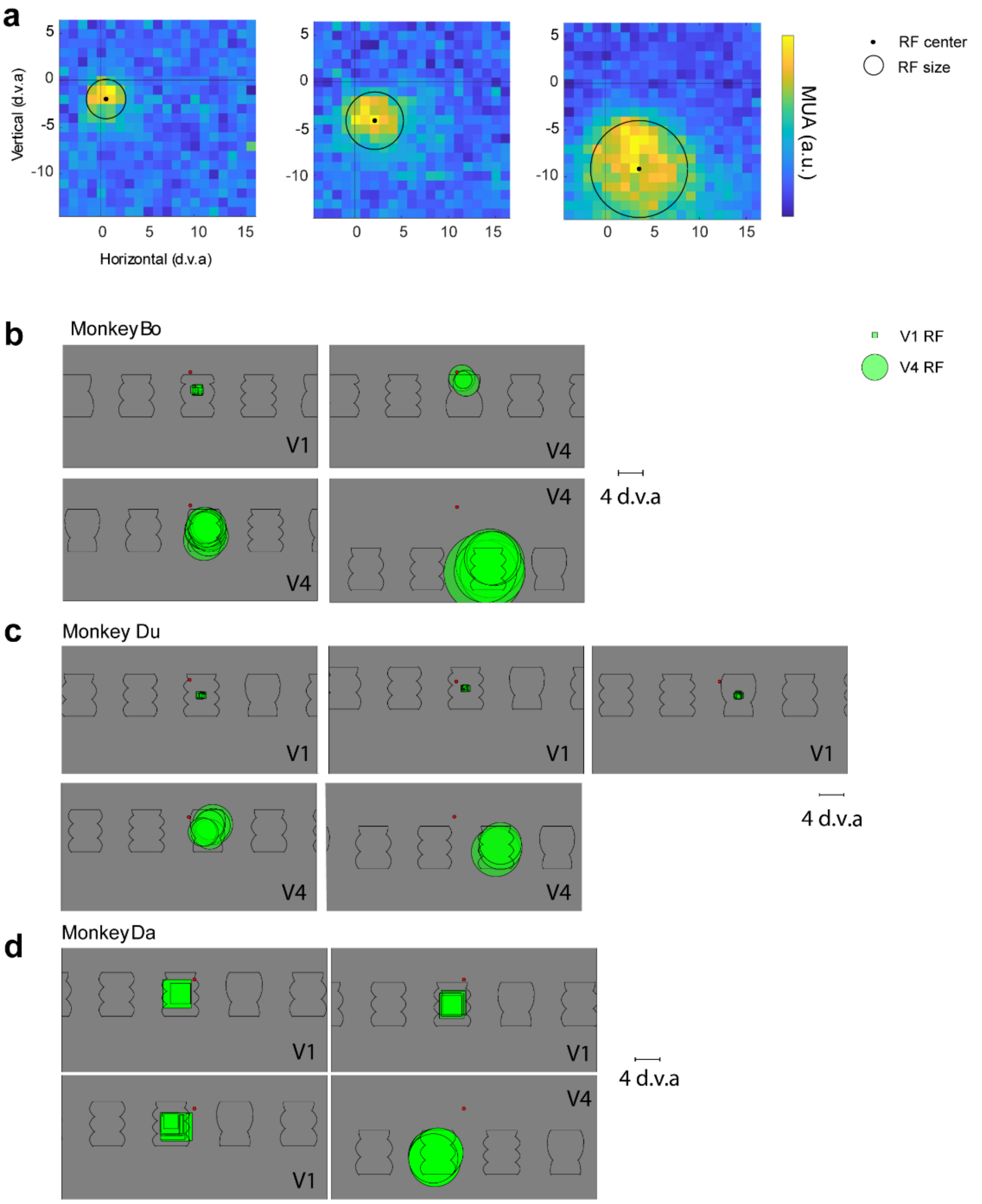

Figure S1. Receptive field sizes and locations. a) Example V4 MUA RFs from electrodes situated on three different Utah arrays. The size of the RF (black circle) and the location of the center (small black dot) were estimated by fitting a 2D Gaussian. Fixation is at (0,0), presented by the thin blue lines crossing each other. **b**) RFs of monkey Bo per array: individual V1 RFs are depicted by green squares and individual V4 RFs by green circles. RFs of V1 electrodes were mapped with a moving bar stimulus and those of V4 electrodes with flashing squares. Aggregate RFs were calculated by taking the averages of the center coordinates of the individual RFs. **c,d**) Same as a, but for monkey Du (c) and Da (d).

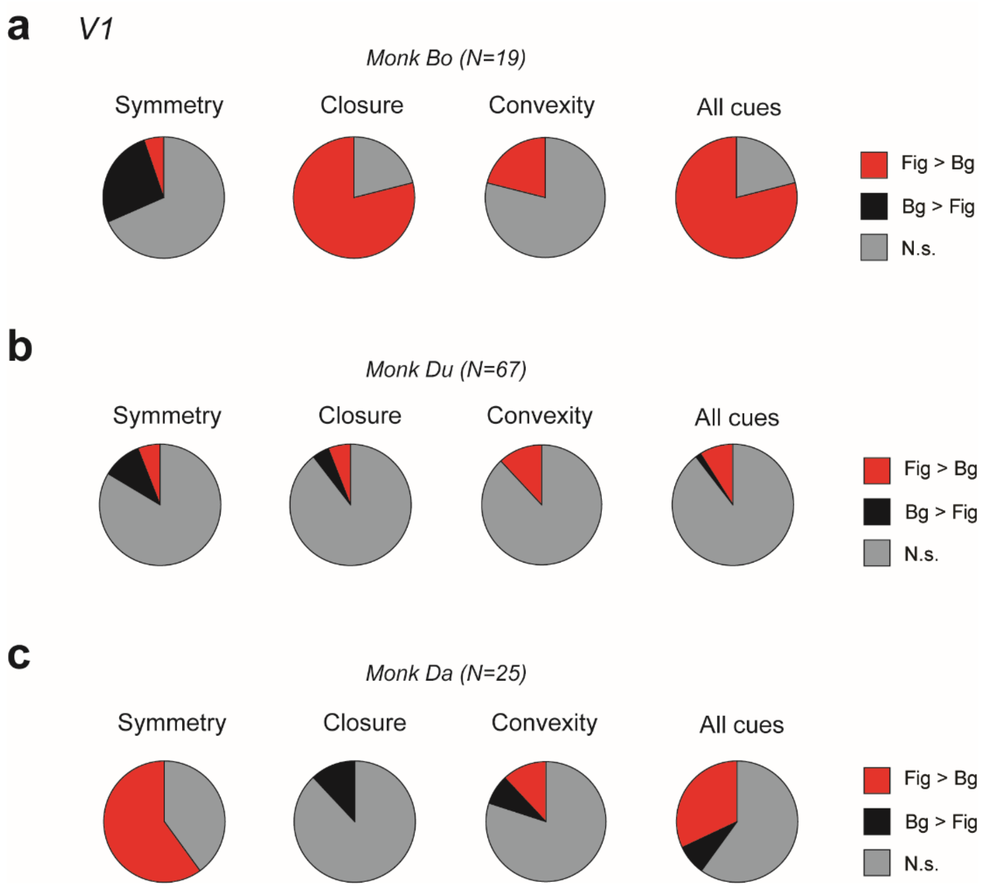

Figure S2. Distribution of significant figure-background effects across V1 recording sites in the three animals. a) The proportion of V1 sites in monkey Bo with a higher response to the figure (red, $p<0.05$) or to the background (black), per Gestalt cue. Sites without a significant difference are shown in grey. **b,c**) Same as a, but in monkey Du (b) and monkey Da (c).

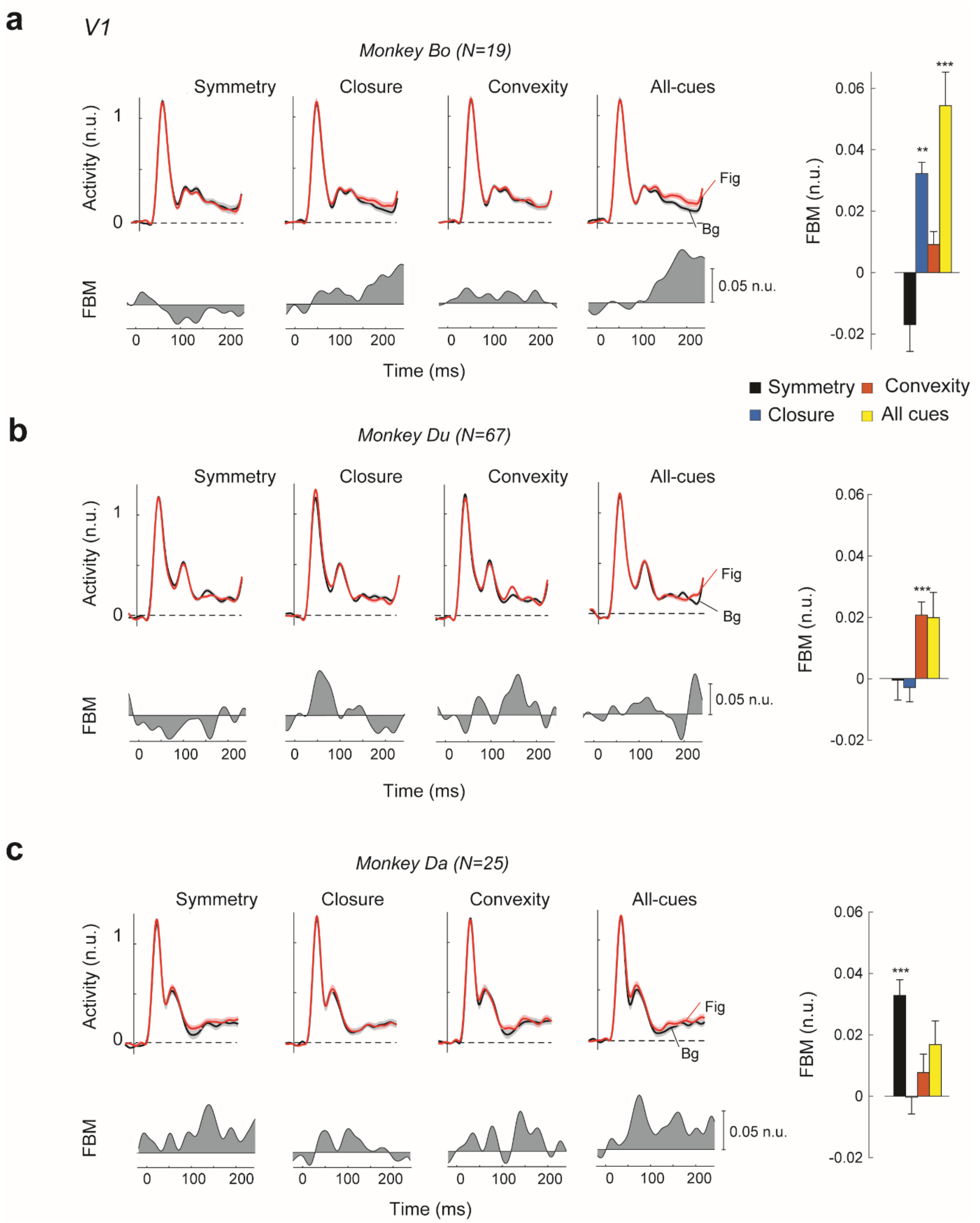

Figure S3. Averages across V1 recording sites in the three animals. **a**) Left panel: Average neuronal activity across V1 recording sites from monkey Bo in response to Gestalt-cue defined figures (red) and backgrounds (black). The lower panel shows the difference in neuronal activity between figures and grounds (FBM). Right panel: Average FBM for the different cues (symmetry = black, convexity = orange, closure = blue, All cues = yellow). Error bars indicate s.e.m. *** = p<0.001, ** = p < 0.01, * = p < 0.05 (All Bonferroni corrected). **b, c**) Same as a, but from monkey Du (b) and monkey Da (c).

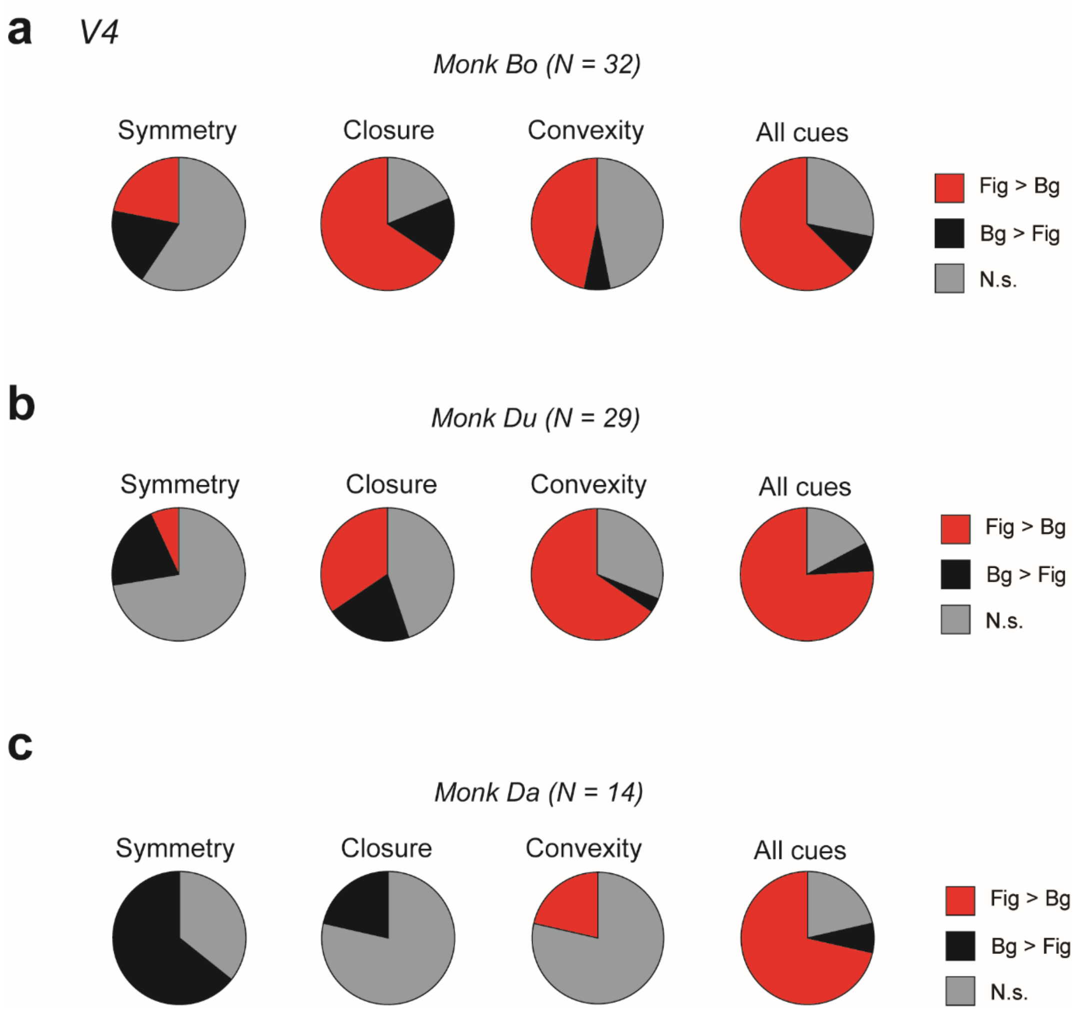

Figure S4. Distribution of significant figure-background effects across V4 recording sites in the three animals. a) The proportion of V4 sites in monkey Bo with a higher response to the figure (red, $p<0.05$) or backgrounds (black), per Gestalt cue. Sites without significant difference are shown in grey. **b,c**) Same as a, but in monkey Du (b) and monkey Da (c).

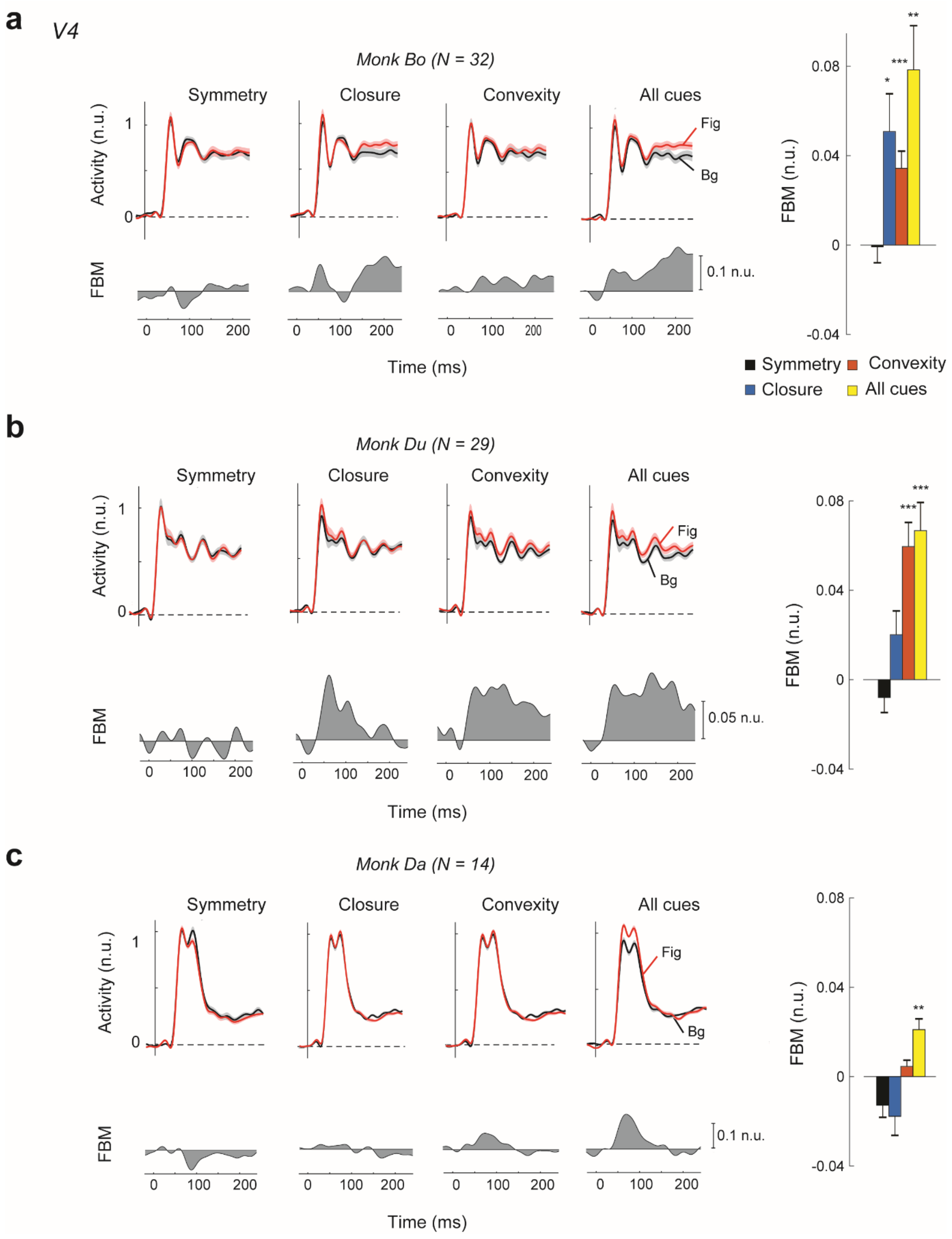

Figure S5. Average responses in V4 from individual monkeys. a) Left panel: Average neuronal activity across V4 recording sites in monkey Bo in response to Gestalt-cue defined figures (red) and backgrounds (black). The lower panels show FBM, which is the difference in neuronal activity between figures and grounds. Right panel: Average FBM for the different cues (symmetry = black, convexity = orange, closure = blue, All cues = yellow). Error bars indicate s.e.m. *** = $p<0.001$, ** = $p < 0.01$, * = $p < 0.05$ (All Bonferroni corrected). **b,c**) Same as a, but in monkey Du (b) and monkey Da (c).

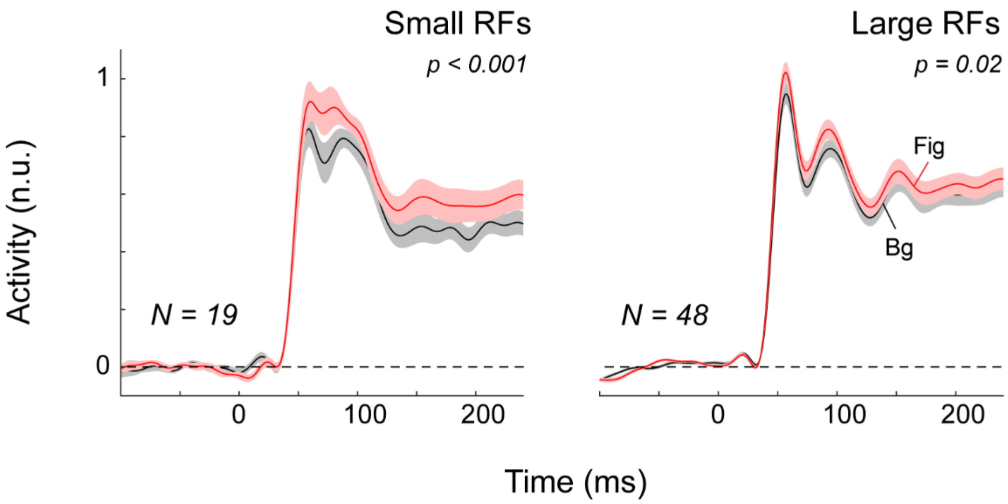

Figure S6. V4 sites with small RFs have stronger FBM. Population responses to figures (red) and backgrounds (black) from V4 from the All-cues condition. We separately analyzed the activity of recording sites with RFs that did and did not overlap with the boundary between the image regions. Small RFs (FWHM < 4º) did not include the border whereas large RFs did have overlap with the border. The p-values are from paired t-tests performed separately for each group.

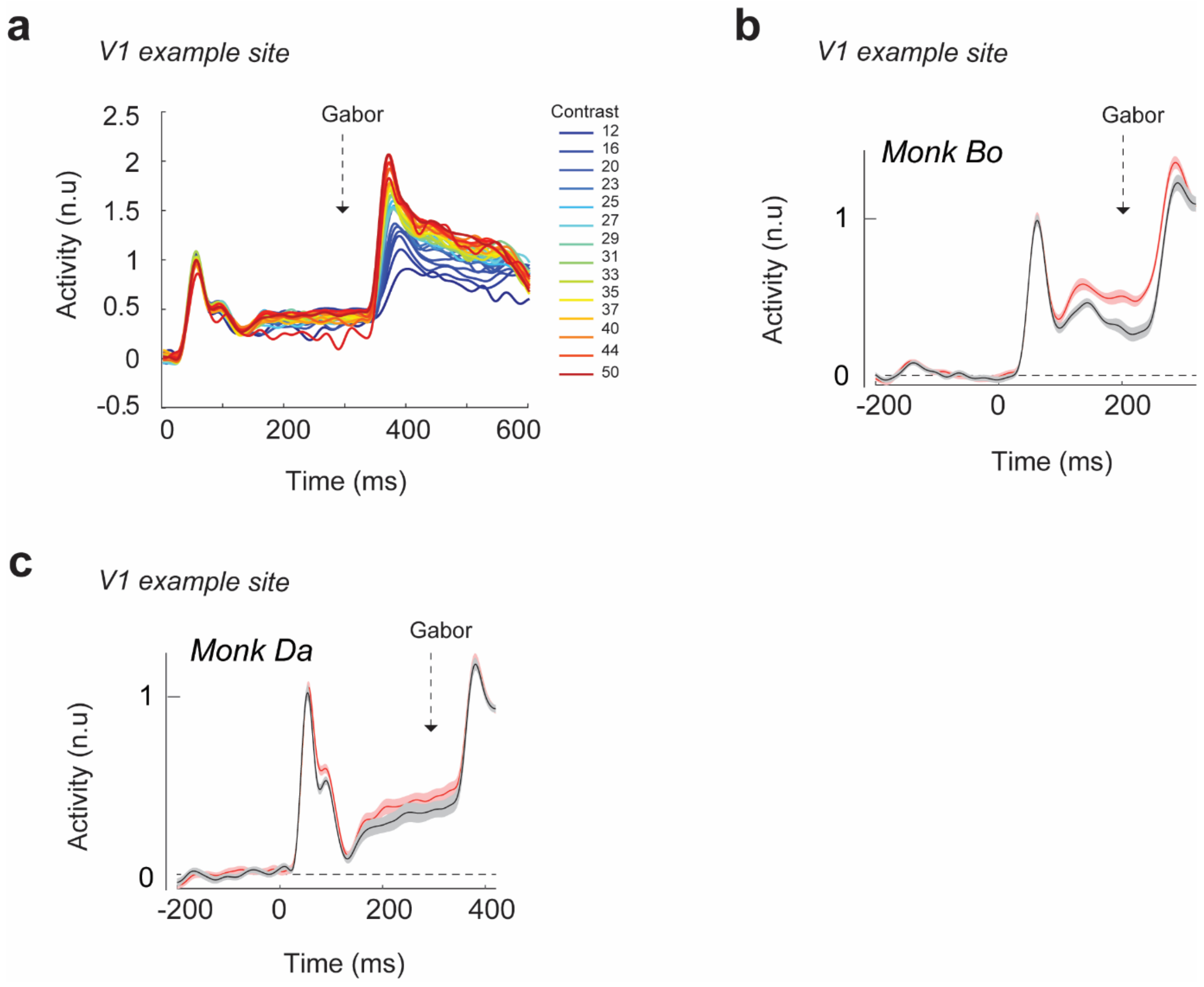

Figure S7. V1 Gabor response. a) The responses of neurons at an example V1 recording site to test Gabors with different contrast levels. The test Gabor was always shown on the ambiguous texture strip. Test Gabor stimuli of higher contrasts elicited stronger transient responses than those with lower contrasts and at a shorter latency. **b**) V1 example site from monkey Bo, showing figure and ground responses over time. The Gabor appeared at 200ms. **c**) Same as b, but for monkey Da. The Gabor appeared at 300ms.

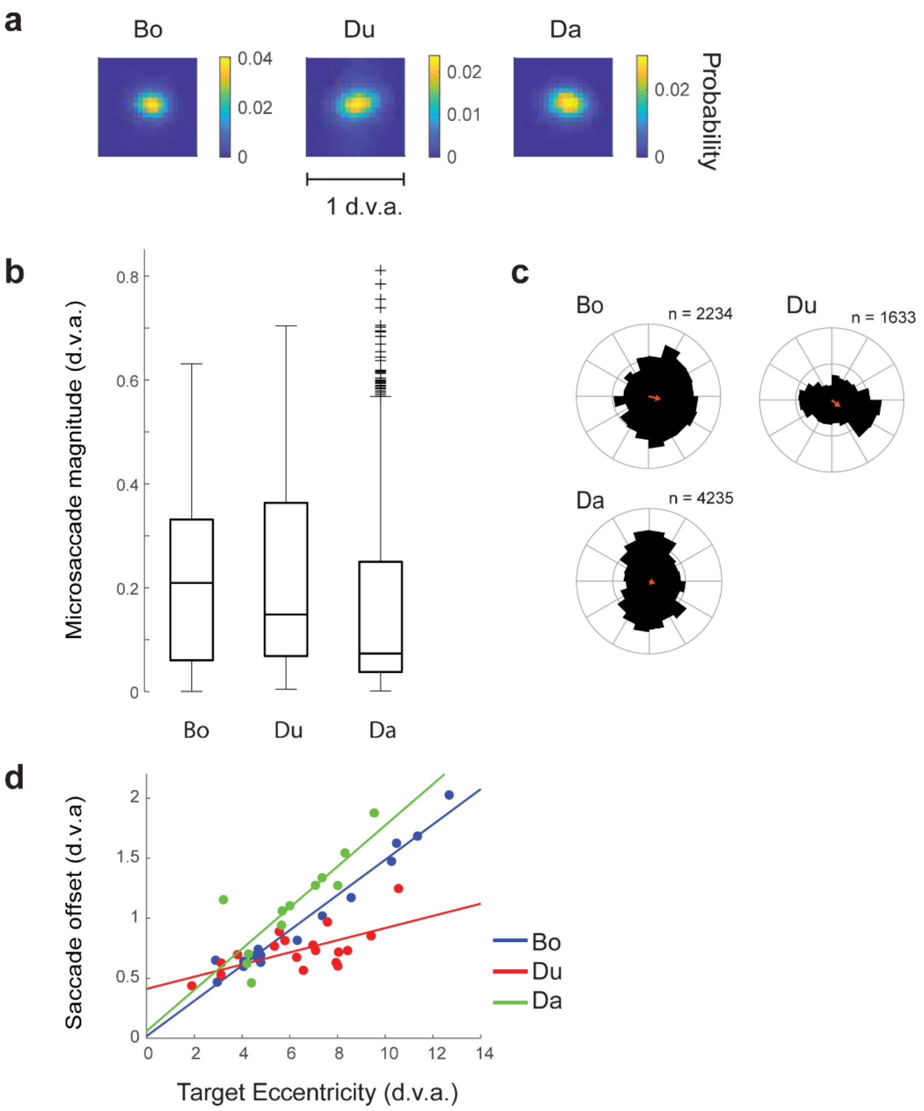

Figure S8. Analysis of micro-saccades and saccadic accuracy. a) Fixation accuracy of monkey Bo (left), monkey Du (middle) and monkey Da (right). The density plots show the probability of the average gaze direction in the window of 0-200ms falling withing a particular 2D bin. Fixation behavior was accurate and well within the fixation window of 1 d.v.a. for each of the monkeys. **b**) Micro-saccade magnitude for monkey Bo (left), monkey Du (middle) and monkey Da (right). Micro-saccades of monkey Da were smaller than those of monkey Bo and monkey Du. **c**) Histograms showing the direction of microsaccades. Monkey Da's microsaccades tended to be more directed along the vertical axis, and those of monkey Bo and monkey Du tended to be directed towards the right, which is the hemi-field in which the Gabor targets were presented for these monkeys. The red arrow shows the average micro-saccadic vector. **d**) Relationship between saccadic accuracy, defined as the Euclidean distance between the saccadic landing point and the center of the target window, and target eccentricity. The saccades were generally accurate, with an expected decrease in accuracy at higher target eccentricities.
